# *In vitro* coronal protein signatures and biological impact of silver nanoparticles synthesized with different natural polymers as capping agents†

**DOI:** 10.1039/d0na01013h

**Published:** 2021-05-17

**Authors:** Priyanka Srivastava, Cindy Gunawan, Alexander Soeriyadi, Rose Amal, Kyle Hoehn, Christopher Marquis

**Affiliations:** School of Biotechnology and Biomolecular Sciences, University of New South Wales Sydney NSW 2052 Australia c.marquis@unsw.edu.au; School of Chemistry, University of New South Wales Sydney NSW 2052 Australia; School of Chemical Engineering, University of New South Wales Sydney NSW 2052 Australia; School of Biosciences and Technology, Vellore Institute of Technology Vellore Tamil Nadu 632014 India priyanka@vit.ac.in; i3 Institute, University of Technology Sydney NSW 2006 Australia; Mochtar Riady Institute for Nanotechnology Tangerang 15810 Indonesia

## Abstract

Biopolymer-capped particles, sodium alginate-, gelatin- and reconstituted silk fibroin-capped nanosilver (AgNPs), were synthesized with an intention to study, simultaneously, their *in vitro* and *in vivo* haemocompatibility, one of the major safety factors in biomedical applications. Solid state characterization showed formation of spherical nanoparticles with 5 to 30 nm primary sizes (transmission electron microscopy) and X-ray photoelectron spectroscopy analysis of particles confirmed silver bonding with the biopolymer moieties. The degree of aggregation of the biopolymer-capped AgNPs in the synthesis medium (ultrapure water) is relatively low, with comparable hydrodynamic size with those of the control citrate-stabilized NPs, and remained relatively unchanged even after 6 weeks. The polymer-capped nanoparticles showed different degrees of aggregation in biologically relevant media – PBS (pH 7.4) and 2% human blood plasma – with citrate- (control) and alginate-capped particles showing the highest aggregation, while gelatin- and silk fibroin-capped particles revealed better stability and less aggregation in these media. *In vitro* cytotoxicity studies revealed that the polymer-capped particles exhibited both concentration and (hydrodynamic) size-dependent haemolytic activity, the extent of which was higher (up to 100% in some cases) in collected whole blood samples of healthy human volunteers when compared to that in the washed erythrocytes. This difference is thought to result from the detected protein corona formation on the nanoparticle surface in the whole blood system, which was associated with reduced particle aggregation, causing more severe cytotoxic effects. At the tested particle concentration range *in vitro*, we observed a negligible haemolysis effect *in vivo* (Balb/c mice). Polymer-capped particles did accumulate in organs, with the highest levels detected in the liver (up to 422 μg per g tissue), yet no adverse behavioural effects were observed in the mice during the duration of the nanoparticle exposure.

## Abstract 2

Biopolymer-capped silver nanoparticle synthesis. Compositional and stability analysis of synthesised particles. Proteomic analysis of particles following serum exposure. *In vitro* hemolytic assays. Organ distribution following administration in mice.

## Graphical abstract

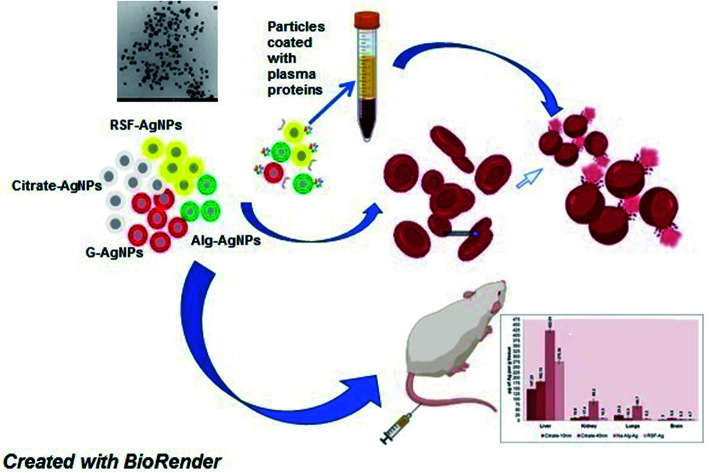

## 1. Introduction

Nanosilver (AgNPs) has been extensively used in a variety of biomedical applications due to its potent antimicrobial effects. Applications include as coatings on implants and surgical instruments, as embeddings in wound dressings and bone substitute biomaterials, the latter such as silver-containing calcium phosphate cements, in contraceptive devices^1^ as well as dental implants,^2^ and to fight bacterial infections. Silver nanoparticles have also been incorporated in burn healing ointments and quite recently, in surgical antibacterial sutures.^3^ With the many applications, it is therefore important to assess the safety of AgNPs in humans. Elevated blood plasma and urine silver concentrations, for example, have been reported in burn wound dressing applications of AgNPs.^4,5^ Relevant to their biomedical applications, the high surface area to volume ratio of AgNPs enables these particles to cross cell membranes, interacting with various biomolecules and cellular components.^6^ Some of the major effects of AgNPs are indeed on the membranes and metabolic activity of human cells, as well as inflicting a myriad of secondary effects, such as generation of reactive oxygen species (ROS) and damage to DNA.^7^

The use of natural biopolymers for the synthesis and stabilization of nanoparticles, or in other words, as capping agents, has gained more attention in recent years due to the anticipated nanoparticle–polymer conjugate compatibility for biomedical applications. The present work synthesized biopolymer-capped AgNPs: alginate-Ag, gelatin-Ag and silk fibroin-Ag, and studied their *in vitro* and *in vivo* cytotoxicity. These biopolymers are among the major ones currently being developed. Alginate is a natural anionic polysaccharide found in seaweed, which is composed of β-(1,4) linked d-mannuronic acid and α-l-guluronic acid units.^8^ Gelatin is a heat-denatured protein (a collagen) and contains many glycine (almost one in every three residues), proline and 4-hydroxyproline amino acid residues (a typical structure would be Ala–Gly–Pro–Arg–Gly–Glu–4Hyp–Gly–Pro).^9,10^ Silks are proteins that are present in the glands of arthropods such as silkworms and bees, and then spun into fibres during metamorphosis.^11^ Silk from *Bombyx mori* (silk worm) consists of two main proteins, sericin and fibroin, with the latter being more commonly used as a nanoparticle capping agent, which is largely made up of the peptide Gly–Ser–Gly–Ala–Gly–Ala.^10,12^ Alginate, gelatin and silk fibroin have been shown to be biodegradable^8–14^ and relatively “invisible” to the immune system.^15^

Scholarly studies have investigated the biomedical applications of alginate-, gelatin- and silk fibroin-Ag nanoparticles and coatings, in particular due to their antimicrobial potential. These nanoparticles were synthesized using various biological approaches, for example *via* the use of plants/plant-derived extracts or their derivatives and microbes, as well as chemical approaches, the main one being the reduction of metallic salts by sodium borohydride.^16–18^ Sharma *et al.*^19^ reported the antimicrobial efficacy of sodium alginate–chitosan–Ag composite films on Gram-positive and Gram-negative bacteria. In another study, Xiang *et al.*^20^ observed the anti-fungal activity of alginate-Ag nanoparticles with higher cell membrane penetrating ability when compared to the bare, non-capped AgNPs. Similarly, for gelatin-Ag, Kubendiran *et al.*^21^ reported higher antimicrobial efficacy of the nanoparticles than those of non-capped AgNPs. Only a few reports have been published on silk fibroin-Ag nanoparticles.^22,23^ Fei *et al.*^22^ observed that silver particle and silk fibroin nanocomposites were effective towards methicillin resistant *S. aureus* and inhibited biofilm formation by the same bacteria. Shivananda *et al.*^23^ reported the synthesis and characterization of silk fibroin and silver nanoparticle composite films. Research inquiries have also studied the antimicrobial activity of other biopolymer-capped AgNPs. Travan *et al.*^24^ studied the synthesis and stabilization of silver nanoparticles with a chitosan-derived polysaccharide and found that the nanocomposite displayed bactericidal activity against both Gram-positive and Gram-negative bacteria.

Biopolymer-capped nanoparticles have also been developed for drug delivery applications to move away from issues often encountered with the use of more conventional nanocarriers, such as liposomes. In many cases, these nanocarriers are associated with extensive aggregation, physical and chemical instability (*e.g.* due to hydrolysis and lipid oxidation), and faster elimination rates from blood circulation.^25–27^ The alginate, gelatin and silk fibroin biopolymers have been used as single conjugates or in combination in drug delivery nano-formulations for entrapment or encapsulation of drugs, such as the chemotherapy drug doxorubicin and the anti-inflammatory agent curcumin; the latter is used, for example, in wound healing applications.^27–29^ Studies have indicated that these nano-biopolymer composites offer better stability (less aggregation due to cross-linking), sustained and targeted release of drugs and low cytotoxicity to many cell lines.^27–30^ The use of the composites was also shown to increase the local concentration of the drug at targeted sites, therefore minimizing any unwanted side effects. The use of other biopolymers has also been studied. Boca *et al.*^31^ reported the synthesis and use of chitosan-coated nanoparticles as photothermal agents. The work reported higher cancer cell mortality in the presence of Chit-Ag nanotriangles when compared to the thiolated poly(ethylene) glycol-capped gold nanorods, the latter being a common hyperthermia agent. These conjugates were internalized by the cancer cells, while exhibiting biocompatibility with human embryonic cells.^31^

Highly relevant to the biomedical application of nanoparticles is their exposure to blood. Nanoparticles can enter the bloodstream through intravenous administration as well as systemic absorption from other biomedical uses, including wound dressings and implants, and can then be distributed to organs and tissues.^32,33^ Interaction of nanoparticles with blood is therefore important and haemo-compatibility should be one of the key factors in the design and development of biomedical NPs.^34^ Furthermore, nanoparticles, when exposed to biological fluids, will be immediately decorated with proteins (in this case, the blood plasma proteins). The composition of these surface-bound proteins, referred to as protein coronas, will determine how the cells ‘see’ and interact with the nanoparticles, in turn affecting the biological behaviour of the particles.^35,36^ Studies have described the importance of particle physicochemical characteristics, including size and surface chemistry, in the protein fingerprints of the corona.^36,37^

Research inquiries have indeed investigated the cytotoxicity of biopolymer-capped AgNPs. Khanh *et al.*^38^ studied the *in vitro* effect of gelatin-Ag on fibroblasts and found that the survival of fibroblasts increased considerably and was even higher than that of the control sample, on application of gelatin–curcumin–silver suspensions at 62.5 μL ml^−1^ concentration. In another *in vitro* study, silver nanoparticle-silk fibroin films were found to support the growth and proliferation of osteoblast cells *in vitro* without disruption of the osteogenic differentiation of hMSCs. This study therefore supported the use of AgNP-silk fibroin films in bone tissue engineering.^39^*In vivo* cytotoxicity studies of biopolymer-capped AgNPs is still scarce. One recent *in vivo* investigation by Diniz *et al.*^40^ studied hydrogels of sodium alginate and gelatin with AgNPs and observed antimicrobial activity, which in turn aided healing of cutaneous lesions in rat models. The work found that these composite hydrogels were non-toxic to fibroblast cells *in vitro*. At this stage, there are still very few cytotoxicity studies of nanoparticles in blood, both *in vitro* and *in vivo*. In view of the increasing use of biopolymer-capped nanoparticles, including AgNPs in the biomedical context, and to gain more reliable knowledge regarding their important cytotoxicity assessment, we herein seek to synthesize and carry out simultaneous *in vitro* and *in vivo* studies on the cytotoxicity of (sodium) alginate-Ag, gelatin-Ag and silk fibroin-Ag, to firstly assess their aggregation behaviour in different biologically relevant media, and study the formation of protein coronas and ultimately their *in vitro* and *in vivo* cytotoxicity on red blood cells, as well as accumulation in organs and tissues in mouse models. The study will assess the nanoparticle haemo-compatibility, with focus on the *in vitro* haemolytic activity in human red blood cells (erythrocytes), both in the absence and presence of blood proteins, elucidating the coronal protein profiles and their potential effects on haemolysis, and ultimately, a preliminary study on the *in vivo* haemolysis potential of the nanoparticles as well as the particle distribution profiles (and silver quantification) in major organs in mice; the latter is to gain insights into the organ compatibility of the biopolymer-capped AgNPs. This type of *in vitro* and *in vivo* compatibility study on blood cells and organs, to the best of our knowledge, has not been reported previously.

## 2. Experimental

### 2.1 Materials

Chemicals including silver nitrate (209 139, ≥99% purity), sodium alginate (A0682, low viscosity), and gelatin type A (G1890, gel strength ∼300 g Bloom) were purchased from Sigma-Aldrich. Lithium bromide (≥99% purity) was purchased from Acros Organics. Sodium carbonate was obtained from Gibco Laboratories. Amicon centrifugal filters and a Pur-A-Lyzer maxi dialysis kit were purchased from Millipore and Sigma-Aldrich, respectively. Human blood samples were procured from the Australian Red Cross Blood Service after obtaining ethical approval from the University of NSW (UNSW HCEC no. HC16756). Balb/c mice were obtained from the University of NSW with ethical approval (17/155B).

### 2.2 Methods

#### Preparation of silver nitrate

Analytical grade silver nitrate was dissolved in Milli-Q water (Synergy Millipore unit) to obtain a stock concentration of 0.5 M. This stock was used to prepare various working concentrations for nanoparticle synthesis.

#### Preparation of stabilizers and/or reductants

Sodium alginate solution (0.5% and 1% w/v), gelatin type A solution (1–6% w/v), and silk fibroin solution (1–3% w/v) were prepared in Milli-Q water. Silk fibroin was extracted from the cocoons of the silkworm *Bombyx mori*.^41^ ChemDraw Professional 16.0 was used to draw the chemical structures of the polymers, presented in ESI Fig. S1.† For silk fibroin, briefly, cocoons were cut into small pieces. 5 g of cocoon pieces was boiled in 0.02 M sodium carbonate solution for 30 min. Following this the fibres were filtered out and washed thrice in distilled water and air-dried overnight. Dried fibres were dissolved in 9.3 M lithium bromide solution in a 1 : 4 volumetric ratio, over a period of 3–4 h with stirring, at room temperature. The dissolved silk fibroin (10 mL) was then dialyzed against 1 L Milli-Q water (with 6 changes) over a 48 h period using 10 K MWCO dialysis tubing (manufacturer). To determine the concentration of silk in solution, a small weighing boat was measured, and 0.5 mL of the silk solution was placed in the boat and allowed to dry at 60 °C. Once dry, the weight of the silk was determined and divided by 0.5 mL and the yielded weight per volume (w/v) percentage was calculated. Following this method, we obtained a yield of 3 ± 0.2%.

#### Synthesis of nanoparticles

The silver nanoparticle synthesis procedure using alginate was broadly based on previous work.^19^ For our work, respective solutions of sodium alginate and gelatin were heated to 90 °C with continuous stirring (200–250 rpm), following which a known concentration of silver nitrate solution was added in a 1 : 5 (v/v) ratio. The mixture was stirred at 90 °C for a further 20 min following which no colour change was observed. It was observed that addition of ascorbic acid or soluble starch (20–100 mg) was required to hasten the gelatin stabilized reduction. Silk fibroin stabilized silver nanoparticles were synthesized at 37 °C and 60 °C under static conditions over a 48 h period.

#### Purification and concentration of particles

Differential centrifugation was adopted as a purification strategy. 1 mL of the samples was centrifuged sequentially at 500*g*, 5000*g* and finally 16 000*g* for 20 min at ambient temperature. After each step, the supernatant was transferred to a fresh tube and centrifuged at the next higher speed to obtain relatively monodisperse particle preparation. Amicon centrifugal filters (50 K MWCO, Millipore) and dialysis were further used to remove any leftover unreacted constituents from the mixture and for concentration of nanoparticle suspensions. 5–7 mL of composite solutions was placed in 50 mL Amicon filters and centrifuged at 5000*g* according to the manufacturer's instructions, for 60 min and 75 min for this concentration step. For dialysis, each of the three particles dialysed were placed in Pur-A-Lyzer cassettes against ultrapure water, with sample-to-buffer ratios of 1 : 100 and 1 : 200, for a period of 24 h with five changes of water.

#### Characterization

##### UV-visible spectroscopy

The initial indication of the formation of silver nanoparticles was recorded in a UV-Visible spectrophotometer (SPECTROstar Nano, BMG Labtech) using a scan range of 200–900 nm. Composite solutions were diluted where necessary to prevent deviation from Beer–Lambert's law and obtain reliable values and observance of a peak with an absorption maximum between 390 nm and 450 nm was considered to be the evidence of formation of AgNPs.

##### Dynamic light scattering and zeta potential

The average hydrodynamic diameter and polydispersity index of different AgNPs were determined by dynamic light scattering (DLS) using a Zetasizer Nano ZS (Malvern) after correction for the change in viscosity due to the presence of the high molecular weight polymer in the final dispersion. All the observations were recorded at 25 °C.

##### Transmission electron microscopy

An FEI Tecnai G2 20 TEM with a thermionic source (LaB_6_) was used to determine the morphology and the size of particles. Before taking the images, particles were centrifuged, and the pellet obtained together with a little supernatant was dropped onto a carbon film coated copper grid. Excess solution was removed carefully using Whatman filter paper no. 1. The grid was then allowed to air dry and then mounted on a single-tilt holder and imaged in bright-field.

##### X-ray photoelectron spectroscopy

Surface chemical characterization of the particles was performed on a Thermo ESCALAB 250Xi X-ray photoelectron spectrometer (XPS), equipped with a monochromated Al K alpha (energy 1486.68 eV) X-ray source. The power of the anode was 120 W (13.8 kV × 8.7 mA) and pass energy was set at 100 eV for survey scans, and 20 eV for region scans. The spot size subjected to analysis was 500 μm and photoelectron take-off angle was 90°.

##### Inductively coupled plasma optical emission spectroscopy

ICP-OES (PerkinElmer OPTIMA 7300) was used to calculate the concentration of silver nanoparticles in solution. 20 μL of the sample was digested with 200 μL of conc. nitric acid and the final volume made to 10 mL using ultrapure water before analysis.

#### Preparation of plasma from whole blood samples

A measured volume of 5 mL of the whole blood samples was fractionated at 2500*g* for 10 min under ambient conditions. The top plasma layer was aspirated out carefully avoiding contamination from the buffy coat and erythrocytes, aliquoted and stored at −80 °C until further analysis. For study in blood plasma, plasma samples were thawed and diluted to 2% (v/v) in PBS (pH 7.4). Plasma was incubated with different particles for 3 h at 37 °C. Thereafter the particle–protein complex was retrieved by centrifugation at 2500*g* for 10 min at ambient temperature. Particle pellets were washed with PBS (pH 7.4) thrice.

#### Stability studies in phosphate buffered saline (PBS)

To determine the stability of particles in PBS, 950 μL of PBS (pH 7.4) was added to 50 μL of 200 μg mL^−1^ individual particle suspensions. The suspensions were mixed gently, and readings were recorded on a UV-Vis spectrophotometer in a scan range of 200 nm to 900 nm.

#### Haemolysis assays on washed erythrocytes and whole blood

Tested whole blood was obtained from the Australian Red Cross Blood Service (Sydney). Blood samples were fractionated at 2500*g* for 10 min at ambient temperature. Upon fractionation, plasma was aliquoted and stored separately at −80 °C for further use. The buffy coat was discarded and the erythrocytes were used in this assay. The erythrocytes obtained were washed in sterile saline solution and centrifuged two more times at 2500*g* for 10 min with a wash with saline solution in between. Following this 1 mL of erythrocytes was diluted with 49 mL of Phosphate Buffered Saline (PBS), *i.e.* in a 1 : 50 ratio (v/v). For all the haemolysis experiments, 160 μL of diluted RBCs was aspirated in a V-bottom 96 well plate and the remaining 40 μL was made up with the negative and positive controls and test samples. For conducting haemolysis assays on whole blood, 1 mL of whole blood was diluted with 49 mL of Phosphate Buffered Saline (PBS), *i.e.* in a 1 : 50 ratio. 160 μL of diluted RBCs was aspirated in a V-bottom 96 well plate and the remaining 40 μL was made up with the negative and positive controls and test samples.

#### LC-MS/MS to elucidate the profile of coronal proteins

A 2% blood plasma solution was incubated with 200 μg mL^−1^ of the tested AgNPs for 3 h, in a 1 : 5 (v/v) ratio, at 37 °C. Following incubation, the samples were centrifuged at 16 000*g* for 20 minutes thrice. After each centrifugation step, the particles were washed with PBS. After the final step, the pellet (sedimented particles) was treated with 2% sodium cacodylate buffer to dissociate all proteins bound to particles and the solutions were subjected to LC-MS/MS analysis. Data from all MS/MS samples were analyzed using Mascot (Matrix Science, London, UK; version 2.6.0). Mascot was set up to search the sprot_14_2_18 database (selected for *Homo sapiens*, unknown version, 20 333 entries) assuming the digestion enzyme trypsin. Mascot was searched with a fragment ion mass tolerance of 0.050 Da. Scaffold (version Scaffold_4.8.9, Proteome Software Inc., Portland, OR) was used to validate MS/MS based peptide and protein identifications. Peptide identifications were accepted if they could be established at greater than 95.0% probability by the Peptide Prophet algorithm with Scaffold delta-mass correction.^42^ Protein identifications were accepted if they could be established at greater than 95.0% probability and contained at least 2 identified peptides. Protein probabilities were assigned by the Protein Prophet algorithm.^43^ Proteins that contained similar peptides and could not be differentiated based on MS/MS analysis alone were grouped to satisfy the principles of parsimony.

#### *In vivo* studies with Balb/c mice

Pilot animal studies were performed on 5–6 week old female Balb/c mice sourced from the University of NSW after obtaining approval as per UNSW guidelines (Ethics approval number 17/155B) to evaluate the *in vivo* haemolysis and organ accumulation pattern of nanosilver. Mice were administered with a single dose of 100 μL of silver nanoparticle suspension *via* lateral tail vein injection. Animals were divided into ten groups and each group was assigned three mice (*n* = 3). Out of ten, mice in each of the nine groups were administered 100 μL of one of two concentrations of silver nanoparticles: 2.5 or 10 mg per kg body weight (*i.e.* 50 μg mL^−1^ or 200 μg mL^−1^) of alginate-Ag, silk fibroin-Ag, citrate-stabilised 10 nm, and citrate-stabilised 40 nm particles. The tenth group was the carrier control and was injected only with sterile saline solution. We omitted gelatin-Ag from animal studies as these particles were affected with contamination on storage. For all studies, two out of three mice were sacrificed after the treatment period of 24 h and one from each group was sacrificed on the 8^th^ day. Mice were sacrificed using carbon dioxide in a chamber where the level of CO_2_ was gradually elevated to minimize discomfort to the animals.

Around 0.5 mL of blood was withdrawn in vacutainers with PBS (pH 7.4) from the thoracic cavity of mice treated with particles after sacrificing the animal post treatment period. Haemolysis was observed visually post centrifugation at 2500*g* for 10 min at ambient temperature.

To evaluate the silver content in organs, mice were dissected post-necropsy and organs stored in sodium cacodylate buffer (0.1 M). For ICP-OES analysis (PerkinElmer OPTIMA 7300), samples were digested with nitric acid (5% v/v final concentration). Weights of individual organs were recorded before the analysis in triplicate to calculate the average amount of silver present in each tissue.

### 2.3 Statistical analysis

Each experiment had a minimum of three to five replicates and each experiment had been repeated at least thrice. One-way and two-way ANOVA were applied to see the statistical relevance of the results. In each case, *p* < 0.05 and *p* < 0.01 (for SOD assay) were considered statistically significant.

## 3. Results and discussion

### 3.1 Synthesis and characterization of biopolymer silver nanoparticles

Three biopolymers (sodium alginate (0.5 and 1% w/v), gelatin (1–6% w/v) and silk fibroin (1–3% w/v)) were evaluated for their capacity as reductants and/or stabilizers for the synthesis of silver nanoparticles from silver nitrate. We found that all biopolymers were able to function as reductants and/or stabilizers, generating relatively small and stable silver nanoparticles. The particle formation was first assessed based on visual colour change. For sodium alginate (optimized reaction temperature of 90 °C, alginate as a reductant and stabilizer), stable silver nanoparticles (alginate-Ag) were formed at 0.5% w/v (20 mL) of the polymer and 0.04 M (5 mL) silver nitrate (2 mmol Ag^+^ per g sodium alginate). For gelatin (90 °C optimum reaction temperature, gelatin as a stabilizer and added ascorbic acid as a reductant), stable silver nanoparticles (gelatin-Ag) were formed at 1% w/v (20 mL) of the polymer and 0.01 M (5 mL) silver nitrate (0.25 mmol Ag^+^ per g gelatin). For the alginate and gelatin, a colourless-to-brownish colour change, an indication of Ag^+^ to metallic Ag^0^ reduction, was observed within 5–10 min of silver nitrate addition into the biopolymer solutions. At higher amounts of silver nitrate, formation of silver aggregates was visible with an immediate colour change. Ultimately, for silk fibroin (37 °C optimum reaction temperature, silk fibroin as a reductant and stabilizer), stable silver nanoparticles (silk fibroin-Ag) were formed at 1.5% w/v (10 mL) of the polymer and 0.04 M (5 mL) silver nitrate (1.3 mmol Ag^+^ per g silk protein). The colour change occurred more slowly for the silk-fibroin system, most likely due to the lower reaction temperature, in agreement with earlier studies.^44,45^ Following a purification step (described in the next section), we characterized the physicochemical characteristics of the polymer-capped particles.

**Fig. 1:**
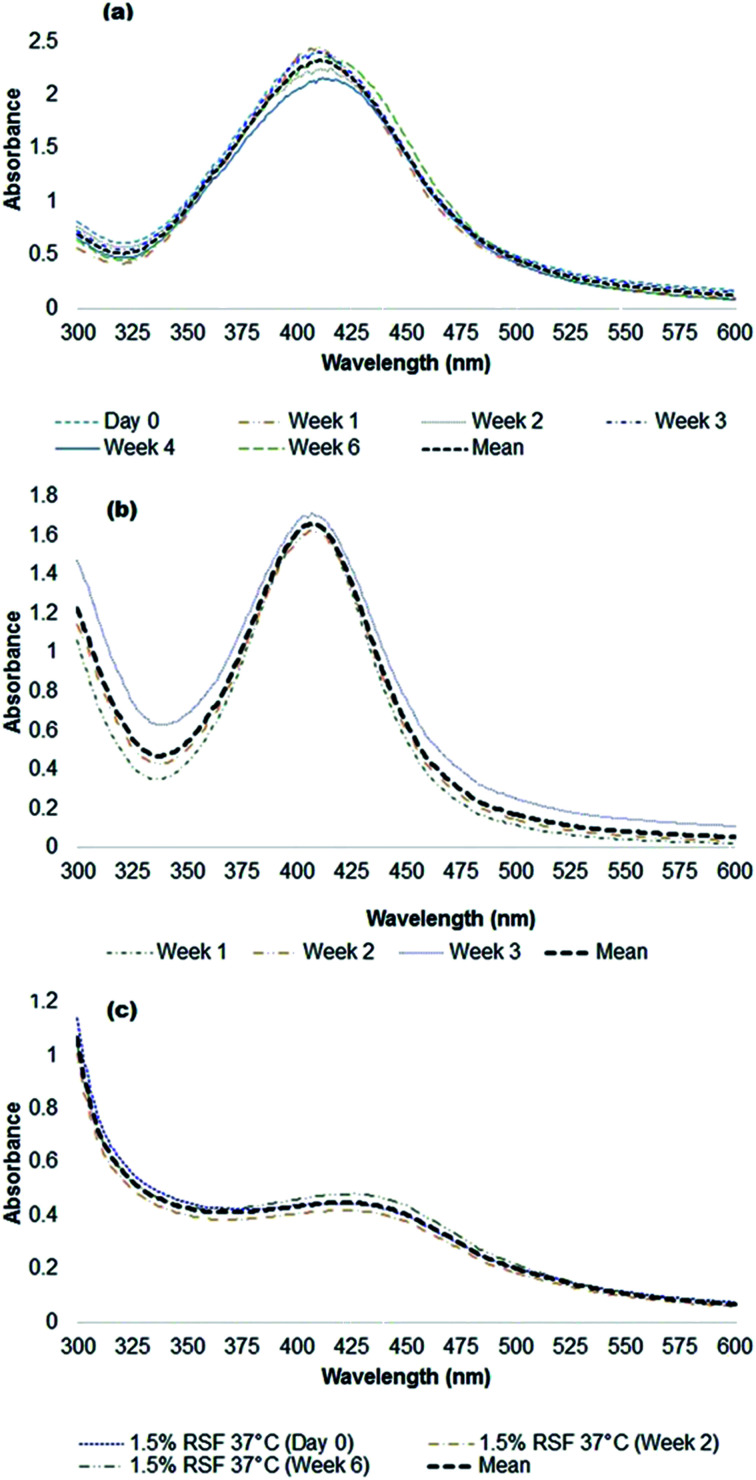
UV-visible spectra of silver nanoparticles in ultrapure water, with (a) sodium alginate, (b) gelatin, and (c) silk fibroin biopolymer cappings. Note that the absorption maxima of the biopolymers do not overlap with those of the silver particles (shown in the figures are the 410–430 nm characteristic localized surface plasmon resonance (SPR) of the particles). Absorption maximum of alginate in water is at ∼210 nm.^74^ Gelatin has two absorption maxima, one corresponds to the presence of peptide bonds and side chains of the aromatic groups (π → π* transition) at 220 nm, and the other corresponds to the aromatic side chains (n → π* transition) at 280 nm.^75^ The absorption maximum of the (unmodified) silk fibroin polymer is at 280 nm.^76^

**Fig. 2:**
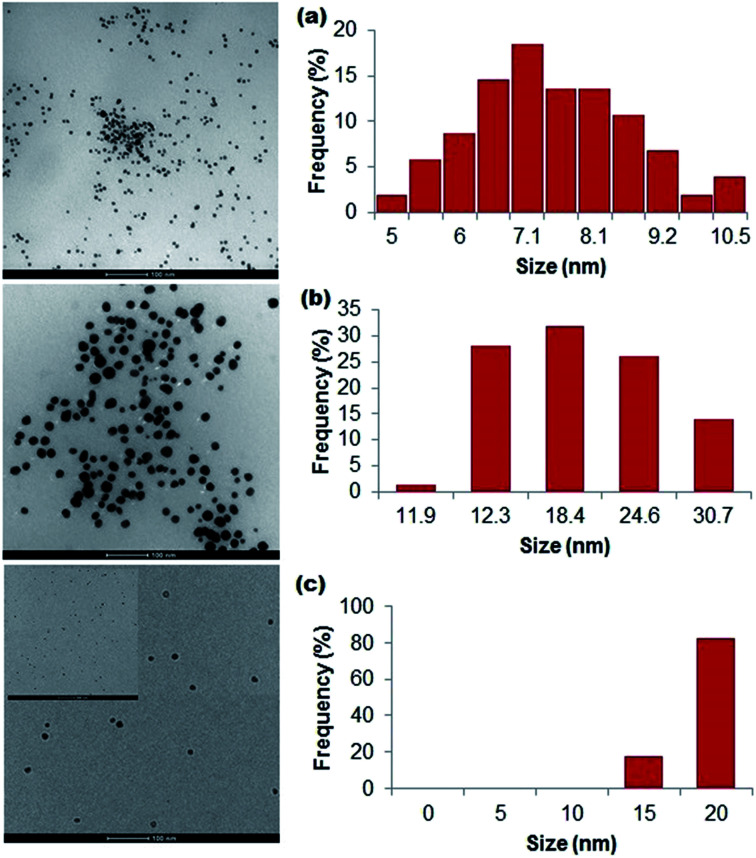
Bright-field TEM images and associated size frequency distribution data of particles stabilized with (a) alginate, (b) gelatin, and (c) silk fibroin, estimated from the images.

Using UV-Vis absorption spectroscopy, a characteristic localized surface plasmon resonance (SPR) was detected at 360–475 nm for all the polymer-capped nanoparticles (Fig. 1), validating the formation of Ag^0^ (silver nanoparticles typically demonstrate SPR at 390 nm to 450 nm).^46^ Detailed spectral analysis of our particles revealed SPR maxima at 410 nm and 409 nm for alginate-Ag and gelatin-Ag, respectively, while a broad SPR hump was detected for silk fibroin-Ag with a 422 nm maximum. A red shift would typically indicate larger particle size;^46^ however further transmission electron microscopy imaging of the particles interestingly showed otherwise: the largest of the polymer-capped particles is the gelatin-Ag (12–30 nm primary size), followed by silk fibroin-Ag (15–20 nm) and alginate-Ag (5–10 nm), all with spherical morphology (Fig. 2). The correlation between the SPR peak position and the particle primary size is complex. For instance, relevant to the polymer-capped particles, the presence of electron donating or withdrawing ligands can shift the SPR peak position relative to the bare particles.^47^ The presence of silver in the polymer-capped nanoparticles was quantified (see Table 1) by inductively coupled plasma optical emission spectrometry (ICP-OES).

**Table 1:** Silver content of the polymer-capped nanoparticles, as determined by ICP-OES

Particles	Silver content (mg mL^−1^)
Na Alg (0.5%)-Ag	1.9 ± 0.02
G (1%)-Ag	0.3 ± 0.05
RSF (1.5%)-Ag at 37 °C	2.3 ± 0.3

**Fig. 3:**
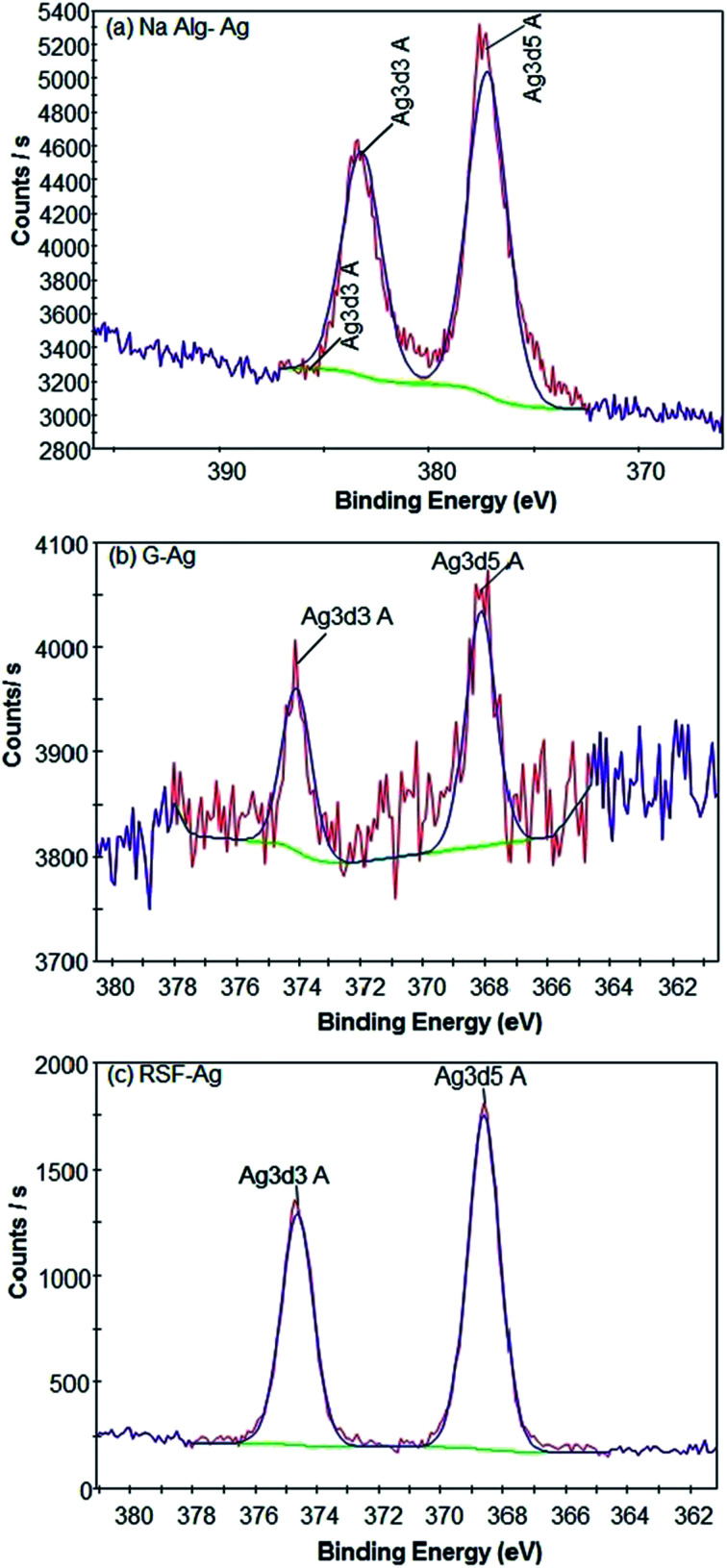
High-resolution XPS spectra indicating the formation of respective silver-biopolymer particles.

We next performed X-ray photoelectron spectroscopy (XPS) to analyse the particles' surface composition. Surface scans showed the presence of Ag, C, O, and N atoms (Fig. 3, ESI Fig. S1 and S2†) in all of the polymer-capped particles, with the specific presence of Na atoms in alginate-Ag, and in gelatin-Ag and silk fibroin-Ag, the presence of S atoms. High resolution scans of the particles further confirmed the presence of the biopolymers on the particles, revealing the chemical bond signatures of the polymers. As presented in Fig. S3,† apart from the expected signature presence of the Na 1s peak in alginate-Ag particles, the scans also showed higher presence of C–O, C–N and C=O relative to C–C bonds, a characteristic of alginate. For gelatin-Ag, the scans showed the signature S 2p peaks, due to the presence of the amino acid cysteine and methionine in the biopolymer. Scans of silk fibroin-Ag showed the presence of an N 1s peak with relatively high binding energy at >406 eV, which is consistent with the N–O interactions in the β-sheet motif of silk fibroin.

### 3.2 Purification of the biopolymer silver nanoparticles

To purify the particle preparations, we assessed two approaches following a three-step differential centrifugation process. The first of the three centrifugation steps (500*g*) removed larger debris, which were present following the synthesis. The second (5000*g*) and third (16 000*g*) steps further removed rather large aggregates that were not visible by the naked eye, yet were clearly seen in microscopic preparations (Fig. S4†). The supernatant from the third step contained a relatively homogeneous and monodisperse population of particles, as evident from the TEM images (Fig. 2). To isolate the particles from unreacted constituents in the supernatant, we assessed two procedures: centrifugation using ultrafilters and dialysis. The centrifugation (5000*g*, 60 min) with the use of a 50 kDa MWCO (molecular weight cut-off) filter was optimal for concentrating the particles, enabling a 10- to 20-fold increase in particle concentration. Centrifugation for longer than 60 min caused further particle aggregation, as well as greater loss of particles with the aggregates adhering on the filter membrane (Fig. S4†). On the other hand, shorter centrifugation of 15 and 30 min led to no or very little supernatant passing through the filter (data not shown). For the alternative dialysis procedure, we observed undesirable sample dilution with all the particle preparations (Fig. S5†). An additional evaporation step is required to concentrate dialysed preparations, which could also lead to unwanted concentrations of any unreacted components within the particle samples.

### 3.3 Stability of biopolymer silver nanoparticles in aqueous environments

**Fig. 4:**
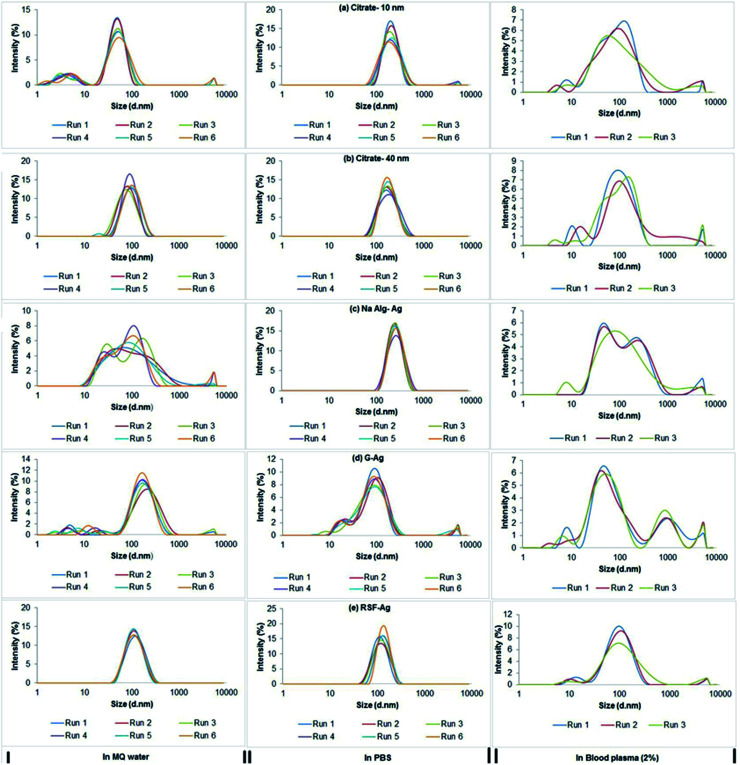
DLS analysis showing intensity *versus* size distribution patterns of particles in Milli-Q water, PBS (pH 7.4) and human blood plasma (2%).

Considering the intended biological use of the biopolymer-capped nanoparticles, we next assessed the hydrodynamic diameter of the particles, that is, their average size when in aqueous systems. Our study also included the commercially purchased citrate-stabilized silver nanoparticles (10 and 40 nm primary size) for size comparisons. Measured using dynamic light scattering analysis, we found that both alginate-Ag (*Z*-ave = 57 ± 2 nm) and gelatin-Ag (59 ± 1 nm) had comparable average size in water, almost half of that of silk fibroin (104 ± 2 nm) (Fig. 4 and Table 2). The biopolymer-capped particles had comparable hydrodynamic sizes to the ‘control’ citrate-stabilized particles, 43 ± 10 nm for the 10 nm particles and 83 ± 11 nm for the 40 nm particles. This relatively low degree of aggregation in water when compared to silver nanoparticles of a similar primary size is most likely due to the presence of functional groups on their surface as stabilizers, which in turn affect the net surface charge of the particles (zeta potential).^48,49^ The zeta potential of alginate-Ag for example is highly negative (−45 ± 2 mV, Table 2), possibly due to the presence of hydroxyl and carboxyl groups in the alginate, facilitating a relatively high degree of electrostatic repulsion between the particles.

**Table 2:** Hydrodynamic diameter and zeta potential values of silver nanoparticles in various media^a^

S. no.	Nanosilver types	Medium	Hydrodynamic diameter, *Z*-ave (*d*, nm)	Polydispersity index	Zeta potential (mV)
1	Citrate-Ag (Sigma 10 nm)	W	43 ± 10	0.3 ± 0.2	−32 ± 3
		PBS	199 ± 11	0.2 ± 0.01	−8 ± 3
		BP	64 ± 1	0.5 ± 0.01	−24 ± 1
2	Citrate-Ag (Sigma 40 nm)	W	83 ± 11	0.2 ± 0.03	−5 ± 0.2
		PBS	166 ± 5	0.1 ± 0.03	−16 ± 1
		BP	82 ± 4	0.5 ± 0.1	−26 ± 1
3	Na alginate (0.5%)-Ag	W	57 ± 2	0.5 ± 0.1	−45 ± 2
		PBS	240 ± 7	0.1 ± 0.01	−24 ± 2
		BP	75 ± 2	0.5 ± 0.03	−27 ± 1
4	Gelatin (1%)-Ag	W	59 ± 1	0.6 ± 0.01	11 ± 2
		PBS	66 ± 6	0.4 ± 0.1	−1 ± 0.5
		BP	57 ± 1	0.7 ± 0.04	−10 ± 0.6
5	RSF (1.5%)-Ag	W	104 ± 2	0.2 ± 0.01	−20 ± 1
		PBS	117 ± 10	0.1 ± 0.03	−5 ± 0.5
		BP	79 ± 5	0.5 ± 0.04	−14 ± 2

a W = Milli-Q water, PBS = phosphate buffered saline (pH 7.4), BP = blood plasma. Values in both tables represent mean ± SD.

Next, we assessed the hydrodynamic sizes of the particles in saline and blood plasma (Fig. 4); both are among the most important aqueous systems associated with biological applications. For example, in drug delivery applications, nanoparticles will first encounter interstitial fluid or blood when delivered intracutaneously or intravenously.^50^ Other relevant cases include implant applications.^51,52^ When suspended in phosphate buffered saline (PBS, pH 7.4), the average hydrodynamic sizes in general increased for all particles, relative to water, indicating aggregation (in particular the alginate-Ag and citrate-Ag with 2- to 4.5-fold larger aggregates), which is consistent with their (nominally) decreasing zeta potential. Aggregation at high salt concentration, in some cases up to 100-fold, has been reported by previous studies, and the phenomenon seemed to be independent of surface modification.^46,48,53^ It is also important to note that in comparison to alginate-Ag, significantly less aggregation was observed for gelatin- and silk fibroin-Ag, the reason for which is still unclear at this stage, as polymers due to their bulky structures have been indicated to exhibit steric hindrance that could limit aggregation of particles.^54–56^ Here in general, the average hydrodynamic size of the biopolymer-capped NPs was much smaller than that of the citrate-Ag ‘control’ particles. This is thought to result from the absence of a polymer layer in the latter, which could be associated with a more significant decrease in the double layer of charge on the nanoparticle surface (the Helmholtz layer), to cause, at least in part, a pronounced increase in aggregation.^56,57^

Interestingly, incubation in blood plasma saw (nominally) increasing zeta potential for all particles, which indeed resulted in a lower degree of aggregation, relative to PBS (in particular the alginate-Ag and citrate-Ag with 2- to 3-fold smaller aggregates). This is most likely to be associated with the formation of a protein corona on the particles due to the presence of proteins in the blood plasma, as next described. Note that in blood plasma, the average hydrodynamic size of all the biopolymer-capped particles was comparable to that of the citrate-Ag particles.

Furthermore, it was found that the particle colloidal suspensions could maintain their size and thus stability (assessed for up to 6 weeks in water, Fig. 1), suggesting that the surface presence of the biopolymers reduces further aggregation of the particles through steric repulsion.^49^ Studies have hypothesized that the likely presence of free polymer chains in the particle systems can sterically hinder the formation of aggregates.^50,51^ The relatively small (57 to 104 nm) size of the polymer-capped particles in aqueous systems, including in a protein-rich environment, and equally importantly, their potential stability in aqueous environments, show the particles' potential for use in biological applications.^52,58^

### 3.4 Elucidation and analysis of protein coronal signatures of particles

**Fig. 5:**
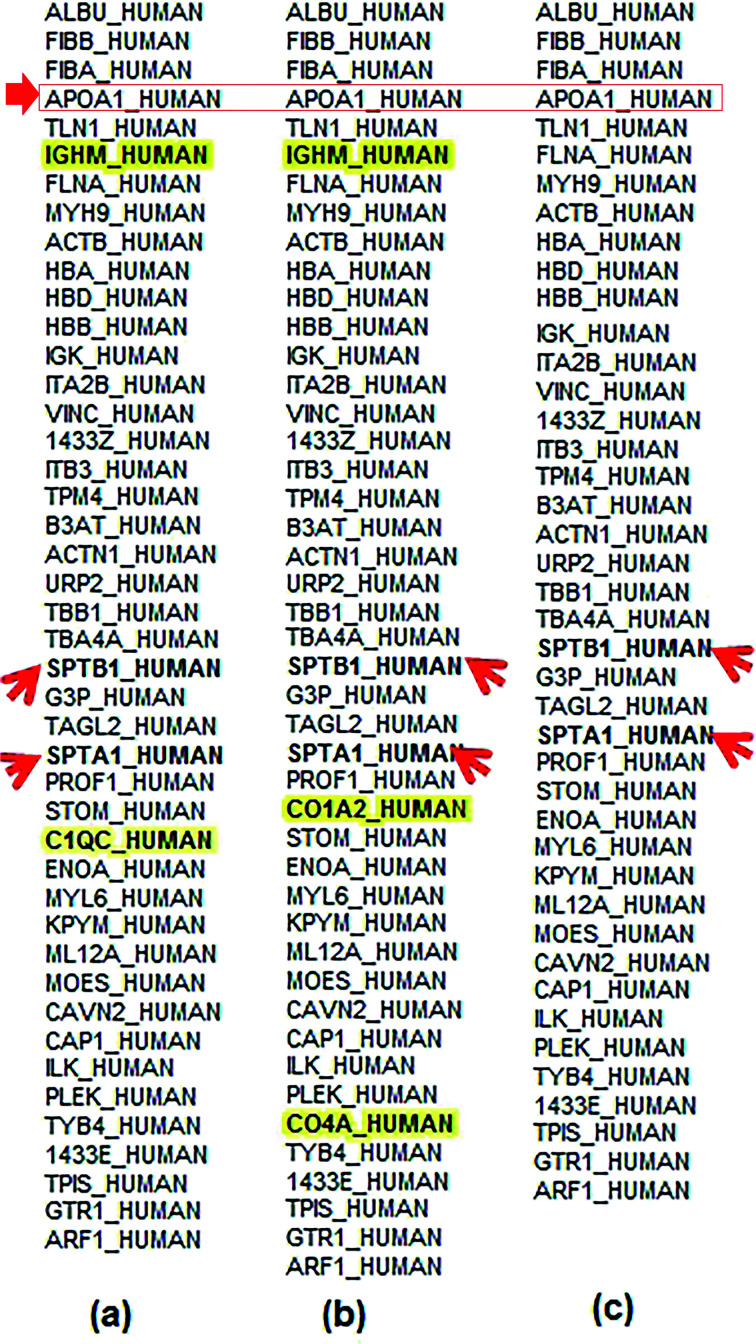
Quantitative profiling, based on average TIC values, of coronal proteins on (a) alginate-, (b) gelatin- and (c) silk fibroin-capped silver particles. The nanoparticles (200 μg mL^−1^) were incubated in blood plasma (2%) for 3 h. The order of appearance of the proteins is based on abundance (with those on the top being the most abundant). The presence of unique proteins in the corona of each particle is highlighted in bold. Arrows show the presence of α- and β-chains of spectrin, as well as apolipoprotein A1 in all the coronas.

Using tandem chromatography-mass spectrometry (LC-MS/MS) analysis, we found 166 proteins adsorbed on the surface of alginate-Ag, 170 proteins on gelatin-Ag and 156 proteins on silk fibroin-Ag. The quantitative profiling of individual proteins is presented in Fig. 5, in order of abundance. The analysis revealed almost identical protein identity for the biopolymer-capped particles. For example, albumin, being the most abundant protein in the plasma, was also the most abundant protein in the coronas of all three particles. Other examples are the presence of alpha and beta fibrinogen, apolipoprotein A1 and talin 1. Earlier studies have indicated that the concentration of proteins in biological fluids could affect the nanoparticle aggregation, being associated with the rate at which the protein corona forms. Less nanoparticle aggregation was seen in fluids with higher protein concentrations (faster rate of protein corona formation), while a higher extent of nanoparticle aggregation were more common in fluids with lower protein concentrations (slower rate of protein corona formation).^59–61^ This is in agreement with the observed smaller hydrodynamic size of our polymer-capped particles in blood plasma, when compared to those in PBS (Table 2).

Although the identity and composition of proteins in the coronas of the polymer-capped particles generated in this study were very similar, we also found the unique presence of a number of proteins in the coronas of different particles. Complement C1q subcomponent subunit C (C1QC) was only detected in the corona of alginate-Ag particles. Likewise, collagen alpha-2(I) chain (CO1A2) and complement C4-A (CO4A) proteins were only found in the corona of gelatin-Ag particles. Immunoglobulin heavy constant mu (IGHM) was found in the corona of both alginate and gelatin-Ag particles, but not in the silk fibroin-Ag corona. The observation of unique protein fingerprints is consistent with many studies, again highlighting the influence of the particles' physicochemical characteristics, in this case, the different sizes and presence of surface functional groups, on the protein identity as well as relative abundance of the formed coronas.^35^ The presence of unique complement proteins like C1QC (on alginate-Ag) and CO4A (on gelatin-Ag) may be due to differences in the surface characteristics like functional groups, curvature and roughness of particles.^36^ According to Gunawan *et al.*,^36^ different sizes and different surfaces are important factors that could lead to preferential adsorption of the complement proteins on certain types of particle and may cause the nanoparticles to induce cell-specific uptake to distinct degrees. Immunoglobulins, on the other hand, are known for their affinity for hydrophobic surfaces.^36^ However, in our study IGHM was found on alginate-Ag and gelatin-Ag and was not present on silk fibroin-capped particles, even though silk fibroin is less hydrophilic than both alginate and gelatin, with long regions of hydrophobic amino acid residues in its structure. This indicates that apart from surface chemistry, other parameters like particle size, curvature and rugosity may have a deciding role in governing protein corona composition. Particles with different coatings show preferential adsorption of specific immunoglobulin types.^36^ Moreover, higher adsorption of IgM relative to IgG on PEG-SWCNTs, in one of the studies, corresponded to lower ratios of spleen *versus* liver accumulation of the particles.^36^ This is in agreement with the observation in our *in vivo* studies. Alginate-Ag particles (IGHM present in the corona) recorded high accumulation in the liver (up to 422 μg Ag per g tissue) of Balb/c, as compared to only up to 275 μg Ag per g tissue in silk fibroin-Ag particles (where no IGHM was detected). Furthermore, we found the presence of erythrocyte cytoskeletal protein spectrin, SPTA1 and SPTB1, in the blood plasma preparations, and interestingly, these proteins were detected in the corona of all the polymer-capped particles (Fig. 5). The findings indicated the affinity of silver particles to the alpha (SPTA1) and beta chains (SPTB1) of spectrin, which could possibly be one of the initial ‘points of interaction’ of silver particles with erythrocytes. However, this needs to be substantiated by further experiments. We next studied the *in vitro* and *in vivo* cytotoxicity of the particles on red blood cells and assessed the *in vivo* distribution of the particles.

### 3.5 Haemolytic activity of biopolymer silver nanoparticles on red blood cells and the effect of protein corona

**Fig. 6:**
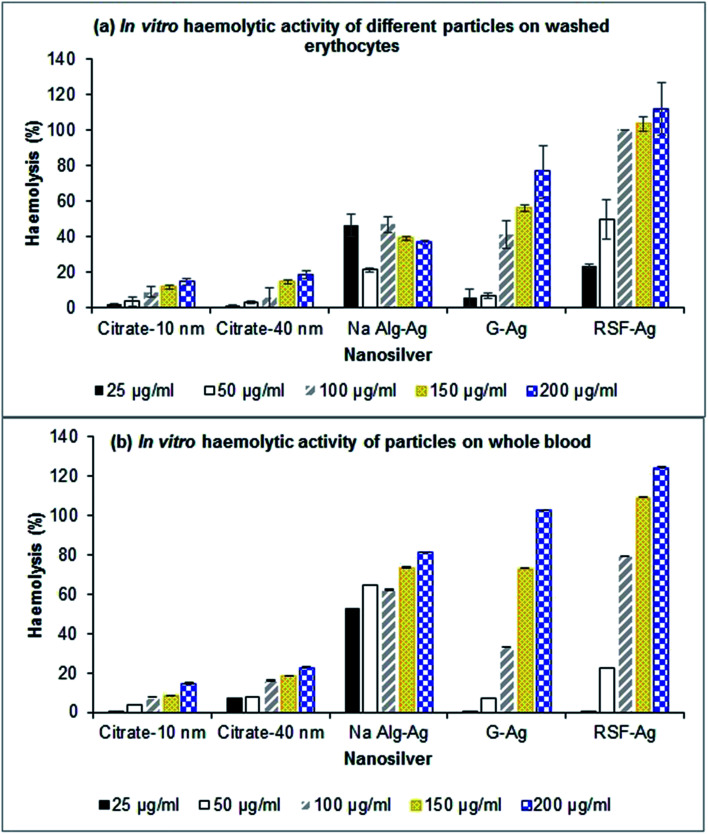
*In vitro* haemolytic activity of various nanoparticles in (a) washed erythrocytes and (b) whole blood. Values are presented as mean ± SD (*n* = 3–5).

The haemolytic activity of the biopolymer-capped particles (alginate-Ag (5–10 nm primary size), gelatin-Ag (12–30 nm) and silk fibroin-Ag (15–20 nm), at 25–200 μg Ag mL^−1^) was studied on washed erythrocytes (red blood cells) and whole blood, the latter to determine the effect of blood plasma proteins on the haemolytic activity. We also included the commercially purchased citrate-stabilized silver nanoparticles of comparable primary sizes with the polymer-capped particles (10 and 40 nm). Erythrocyte lysis was assessed by spectrophotometric measurement of haemoglobin release at 540 nm. For the washed erythrocytes, as shown in Fig. 6a, we observed concentration-dependent haemolysis effects for gelatin-capped and silk fibroin-capped particles, as well as for the citrate-stabilized control particles (note that the haemolytic activity of alginate-Ag was independent of concentration, the reasons for which are unclear at this stage; we have confirmed the observation with multiple experimental replicates). The polymer-capped particles were in general associated with a higher extent of haemolytic activity than the citrate-stabilized particles. Exposure of the washed erythrocytes to 25 to 200 μg Ag per mL polymer-capped particles resulted in 20 to 100% haemolytic activity relative to the positive control (100%) (excluding the gelatin-Ag with ∼5% activity at the lower 25 and 50 μg Ag mL^−1^), while the citrate-stabilized particles were associated with <20% activity for all the tested particle concentrations.

This pattern of haemolytic behaviour most likely relates to the extent of aggregation of the particles. The polymer-capped particles, in particular the gelatin-Ag and silk fibroin-Ag, had smaller hydrodynamic size in PBS (the medium for the haemolytic assay, see Table 2 and Fig. S6†) than the citrate stabilized particles, and are therefore thought to interact more closely with erythrocytes due to their higher specific surface area, leading to extensive haemolysis. Similar observations have also been reported by other studies.^62–64^ Chen *et al.*^62^ for example observed a size-dependent haemolytic effect of AgNPs on red blood cells (from fish), more specifically, ∼60% higher haemolytic activity with the smallest particle (15 nm primary size), in comparison to the larger particles (50 nm and 100 nm primary size) that showed less than ∼10% haemolytic activity. This size-dependent effect was also observed by Choi *et al.*^65^ when studying the haemolytic activity of AgNPs in comparison to their micron-sized counterparts. Alternatively, the red blood cells with their surface presence of proteins can strip off the biopolymer from the nanoparticle surface, and in turn directly interact with AgNPs which increases haemolysis, while not being able to strip off the citrate that has stronger affinity to silver than the cell surface proteins.^66,67^

Next, our study with the whole blood yielded interesting results. We found that the particles' haemolytic activity was in general higher in comparison to those in the washed erythrocyte experiment. As shown in Fig. 6b, the biopolymer-capped particles exhibited 50 to 100% higher haemolytic activity (relative to the positive control) when compared to the washed erythrocyte system, in particular for gelatin-Ag and silk fibroin-Ag, while the citrate-stabilized particles were associated with up to 20% activity. Indeed, as previously described, the particle hydrodynamic size was significantly smaller in the presence of blood proteins (measured in blood plasma), in particular for the alginate-Ag (∼70% reduction in size) and silk fibroin-Ag (30% reduction), in comparison to the PBS system, which is linked to the formation of protein coronas on the particles upon exposure to the blood plasma. Earlier studies have also indicated the effect of protein corona formation on the haemolytic potential of metallic nanoparticles. Saha *et al.*^68^ observed that gold nanoparticles (2 nm core size) functionalized with nine different hydrophobic groups exhibited lower haemolytic activity in the presence of plasma proteins. Interestingly, a group also observed no significant adsorption of blood proteins on gold particles upon incubation in blood.^69^

Taken together, our study interestingly observed a higher extent of *in vitro* haemolytic activity with the biopolymer-capped AgNPs in comparison to the citrate-stabilized AgNPs, both in the absence and presence of blood proteins. The smaller aggregate size of the biopolymer-capped NPs is thought to be the key factor for the extensive haemolysis. It is also apparent that the presence of the biocompatible polymer conjugates did not alleviate the already known haemolysis effect of AgNPs on red blood cells, although at this stage, we are not clear about the underlying causes. In whole blood studies, there have been reports suggesting the role of specific proteins in the corona, such as apolipoprotein A1, which aid nanoparticle interactions with the cell membrane.^70^ Apolipoprotein A1, which is present in the corona of all three particles in this study, has been associated with higher haemolysis earlier^71^ and higher cell contact and internalization.^72^ Apolipoprotein A1 is indeed present in the corona of all three biopolymer-capped AgNPs in our study (Fig. 5) which could be a factor in their higher extent of haemolysis in the whole blood samples when compared to those in the absence of blood proteins (washed erythrocytes). While less evident in specific cases, our study found that the extent of haemolysis was in general concentration-dependent.

### 3.6 *In vivo* haemolytic activity and organ distribution of biopolymer silver nanoparticles

**Fig. 7:**
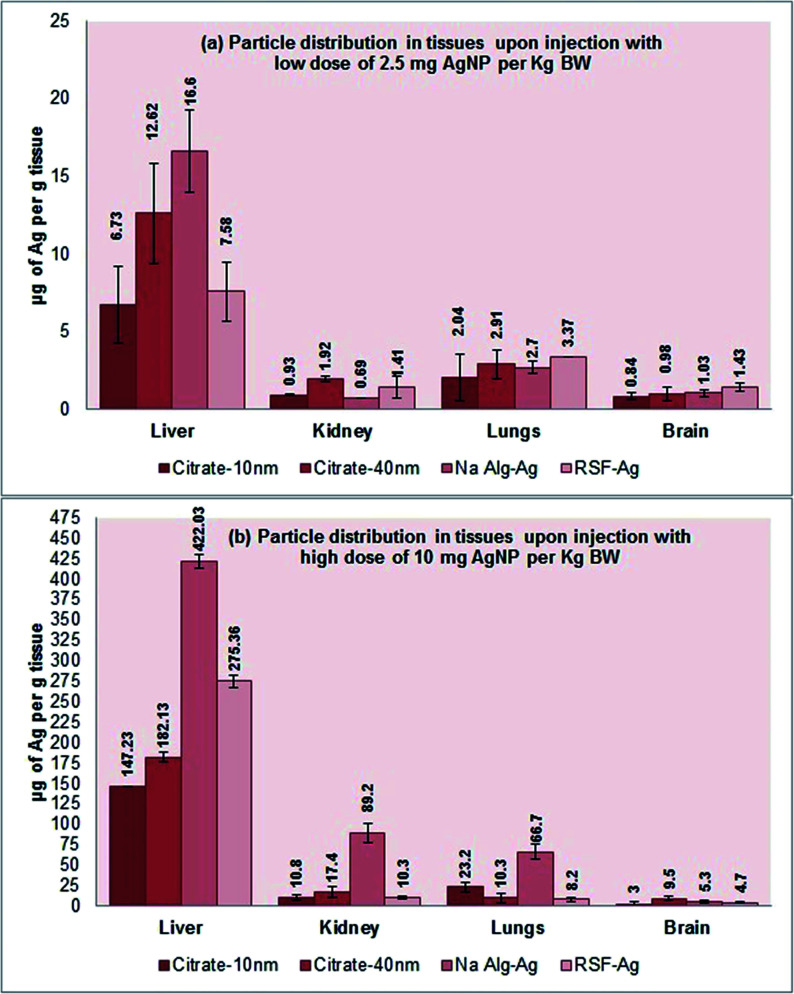
ICP-MS analysis for elucidating the distribution profile of particles in mice organs following intravenous injection: (a) low dose (2.5 mg per kg BW); (b) high dose (10 mg per kg BW).

The study next investigated the haemolytic effects of the biopolymer-capped and citrate-stabilized nanoparticles *in vivo*, as well as their distribution in organs following intravenous dosing. Balb/c mice were intravenously injected with 2.5 or 10 mg per kg body weight (equivalent to 50 or 200 μg mL^−1^ concentration) for 8 days. Following the 8 day exposure, we only detected mild haemolytic activity in the blood for all the tested particles in both low and high dosage groups (Fig. S7†). ICP-OES analysis of tissues revealed the highest silver accumulation in the liver for all particles in both low (6.7 to 17 μg Ag per g tissue) and high (∼150 to 420 μg Ag per g tissue) dosage groups, following 24 h exposure (Fig. 7a and b). This is in agreement with many particle distribution studies, where the presence of fenestrated (porous) endothelium that lines the capillaries in the liver allowing unobstructed flow of plasma from venous blood and permitting liver cells to be ‘soaked’ in plasma is hypothesized as one of the possible reasons for the accumulation of particles in the liver.^73^ Earlier studies by Marchioni *et al.*^73^ detected the presence of Ag_2_S in the liver and had hypothesized the origins of the nano-sized particle accumulation in organs, which could be applicable to the present work. The particles were thought to form from soluble Ag(i) species, either in the organs or in the bloodstream, and then accumulate in organs.^73^ Furthermore, lower presence of silver in the kidneys and lungs was detected for all particles in both low and high dosage groups in our work, with an even smaller presence in the brain. The values in the brain tissue were in the range of 0.8 to 9.5 μg Ag per g tissue (both low and high dosage groups) and are most likely due to the presence of the tightly packed endothelial cells forming the blood–brain barrier, making the barrier almost impermeable, even to nanoparticles. Interestingly, we detected an elevated silver presence in the kidneys and lungs for the alginate-Ag (∼90 and 65 μg Ag per g tissue, respectively) when compared to other particles for the high dosage group. The reason for this remains unclear at this stage. Finally, despite the accumulation of the particles in organs, no adverse reactions (eye squinting, changes in skin color, abnormal movement, limping, unusual feeding patterns and breathlessness) were observed in the mice, even after 8 days of exposure.

## 4. Conclusions

This study successfully synthesized concentrated, well-characterized nanosilver (AgNPs) composite particles with three natural biopolymers – sodium alginate, gelatin type A and silk fibroin. A two-step purification strategy based on sedimentation and ultrafiltration yielded monodisperse particle preparations at 0.3 to 2.3 mg mL^−1^. The biopolymer-capped AgNPs were associated with a relatively low degree of aggregation in water, indicated by their comparable hydrodynamic sizes to those of the control citrate-stabilized AgNPs. Higher degrees of aggregation were in general observed in the biologically relevant medium PBS relative to water for all the biopolymer-capped NPs and the citrate-NPs, while the aggregation reduces in the blood plasma (relative to PBS); the latter is most likely due to the detected formation of the protein corona on the surface of the NPs.

*In vitro* cytotoxicity studies showed a generally concentration-dependent 20 to 100% haemolysis on red blood cells with the biopolymer-NPs, both in the absence and presence of blood proteins. The haemolysis activity was interestingly higher in the presence of blood proteins, which is thought to be associated with the less aggregation of the biopolymer-capped NPs as well as the detected presence of apolipoprotein A1 in their protein corona; the latter is known to increase nanoparticle interactions and uptake by red blood cells and other human cells. The haemolysis activity was also higher when compared to that of the citrate-NPs. Apart from dosage, the effective hydrodynamic size (degree of aggregation) of the biopolymer-capped NPs, which is one of the most important design considerations in biomedical applications of nanoparticles, is apparently the key factor for the observed extensive haemolysis. Any conclusive role of the biopolymer capping agents on the haemolysis is difficult to ascertain as the protein coronas were largely similar in all three particles, with only a few unique proteins on each of them. Our *in vivo* mice studies yielded contrasting results indicating that the biopolymer-capped NPs exhibited only minimal haemolytic activity. Among the studied organs, the liver had the highest accumulation of particles, followed by the lungs, kidneys and brain. Despite the particle accumulation in organs, there was no evidence of adverse effects on the mice.

Overall, the work presented here provided insights into the biological impacts of biopolymer-capped silver nanoparticles, not available previously. Our *in vitro* and *in vivo* studies have raised important research questions that need to be explored to gain an in-depth understanding of the haemocompatibility of natural polymer-capped nanoparticles. In this regard, more extensive, long-term studies are underway to advance our knowledge of particle behaviour, including the origins of the cytotoxicity, to enable unreserved use of these particles in biological applications. Nevertheless, this work has emphasized the importance of carrying out simultaneous *in vitro* and *in vivo* investigations, to formulate reliable and valid conclusions in studies of this nature, that have the potential of being directly implicated in biomedical applications.

## Conflicts of interest

The authors have no conflicts of interest to declare.

## Supplementary Material

NA-003-D0NA01013H-s001

NA-003-D0NA01013H-s002
