# Supplementary material for: In vitro coronal protein signatures and biological impact of silver nanoparticles synthesized with different natural polymers as capping agents
Source: Nanoscale Adv. 2021 May 17;3(15):4424–39. doi: 10.1039/d0na01013h (PMC9418127; doi:10.1039/d0na01013h)
Supplement: NA-003-D0NA01013H-s001 [file NA-003-D0NA01013H-s001.pdf]

Electronic Supplementary Material (ESI) for Nanoscale Advances.
This journal is © The Royal Society of Chemistry 2021

# Synthesis and *in vitro* and *in vivo* comparative investigation of alginate, gelatin and silk-fibroin capped silver nanoparticles

*Priyanka Srivastava[1,4*], Cindy Gunawan[5], Alexander Soeriyadi,[2,6], Rose Amal[3], Kyle Hoehn[1], Christopher Marquis[1*]*

## Supplementary data

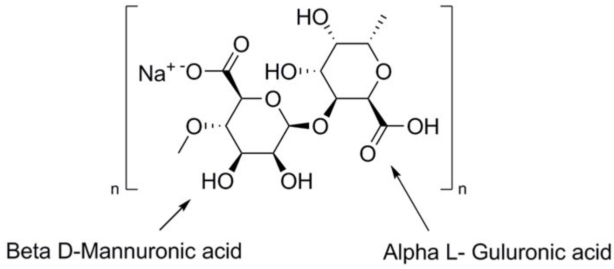


**Sodium alginate derived from Alginic acid**

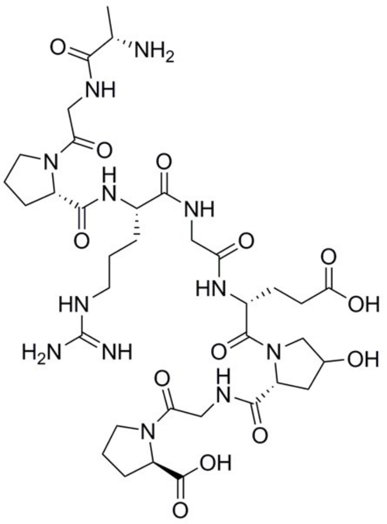

Ala-Gly-Pro-Arg-Gly-Glu-Hyp-Gly-Pro-

**Gelatin**

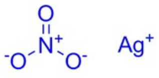

**Silver nitrate**

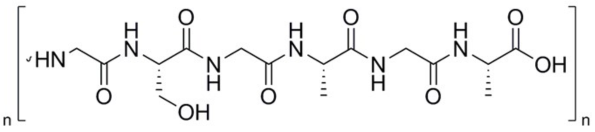

Gly-Ser-Gly-Ala-Gly-Ala

**Silk fibroin**

**Figure S1:** XPS survey spectra of individual atom constituents of a) Sodium alginate b) Gelatin c) Silk fibroin- stabilized nanosilver composites.

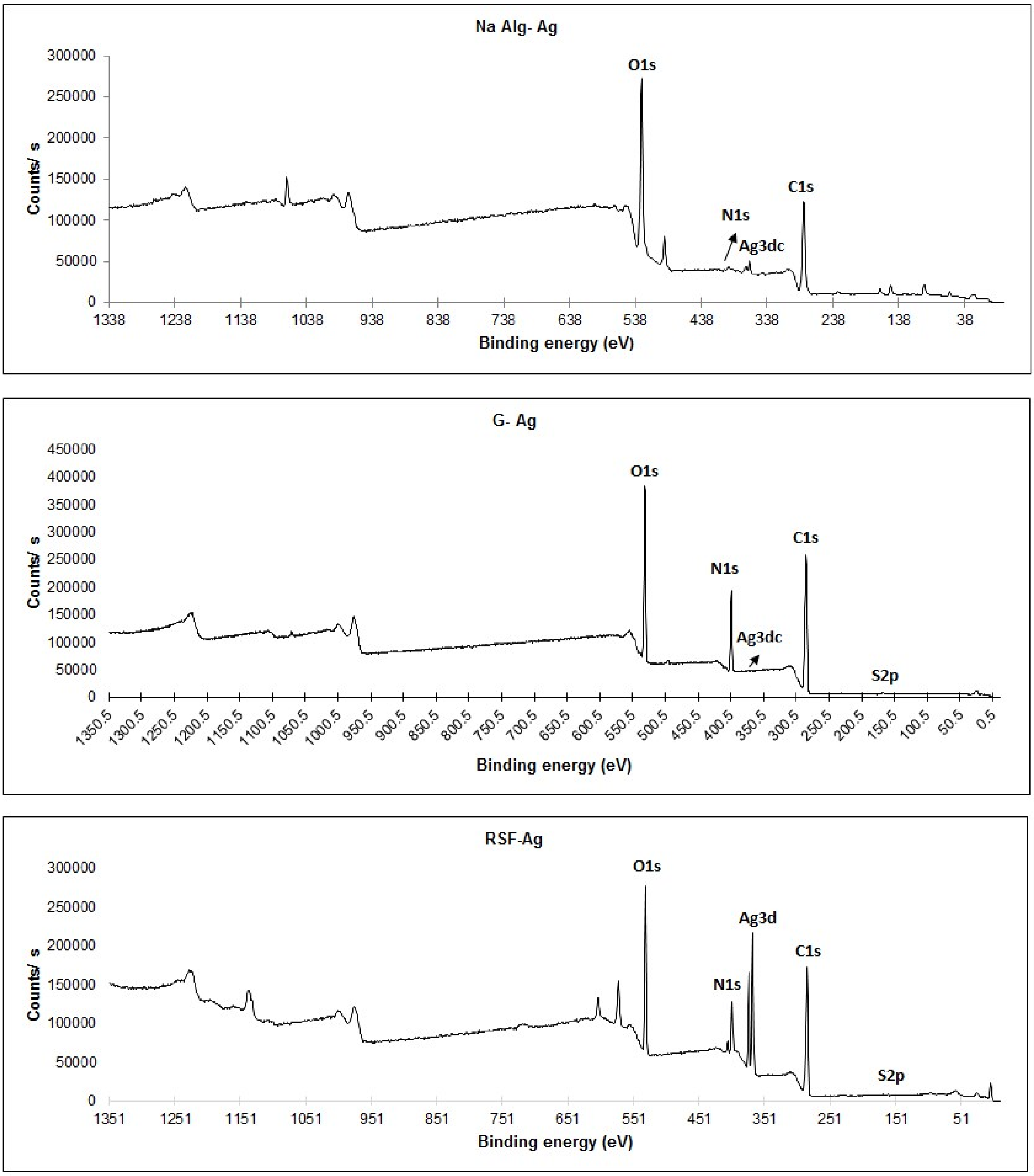


**Figure S2:** XPS survey scans of polymer-silver composites

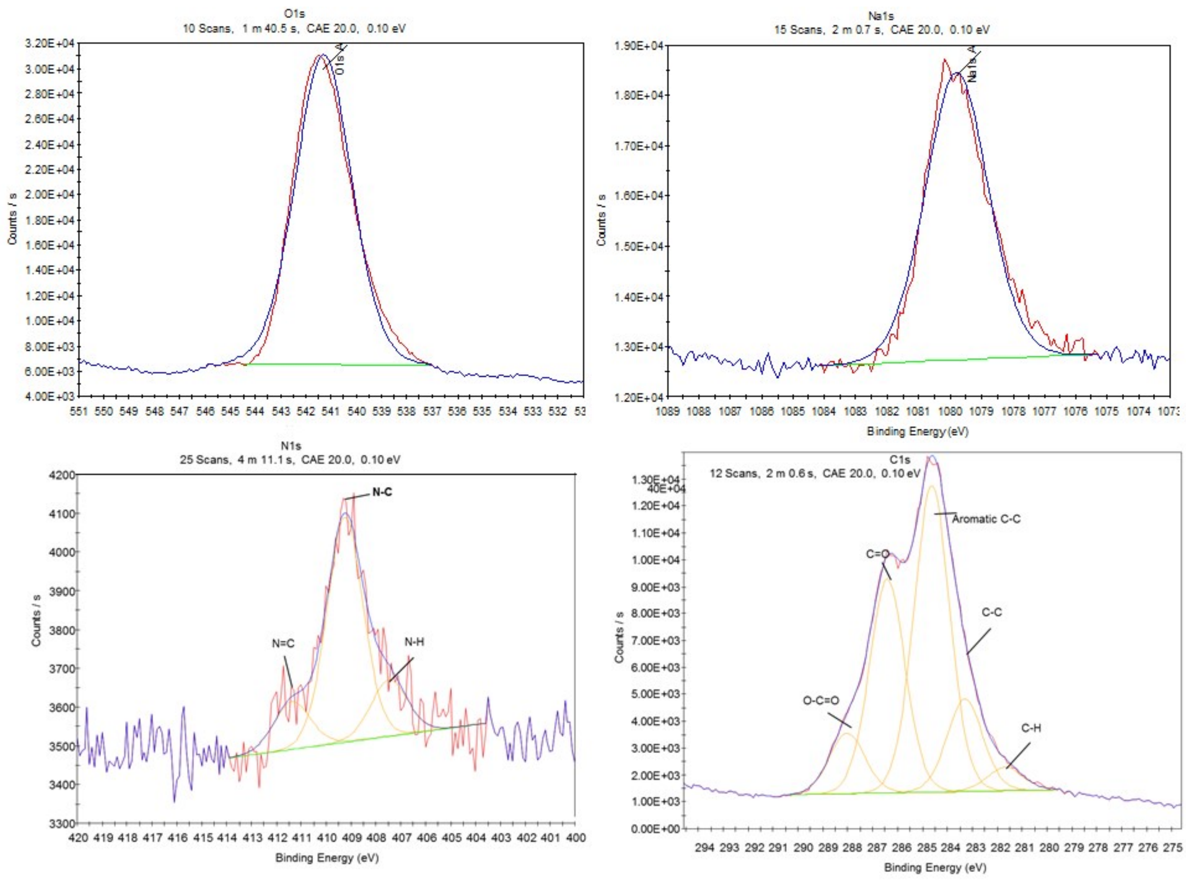


**Figure S3:** High resolution XPS spectra

**(a) Sodium alginate- silver nanocomposites (Na Alg-Ag)**

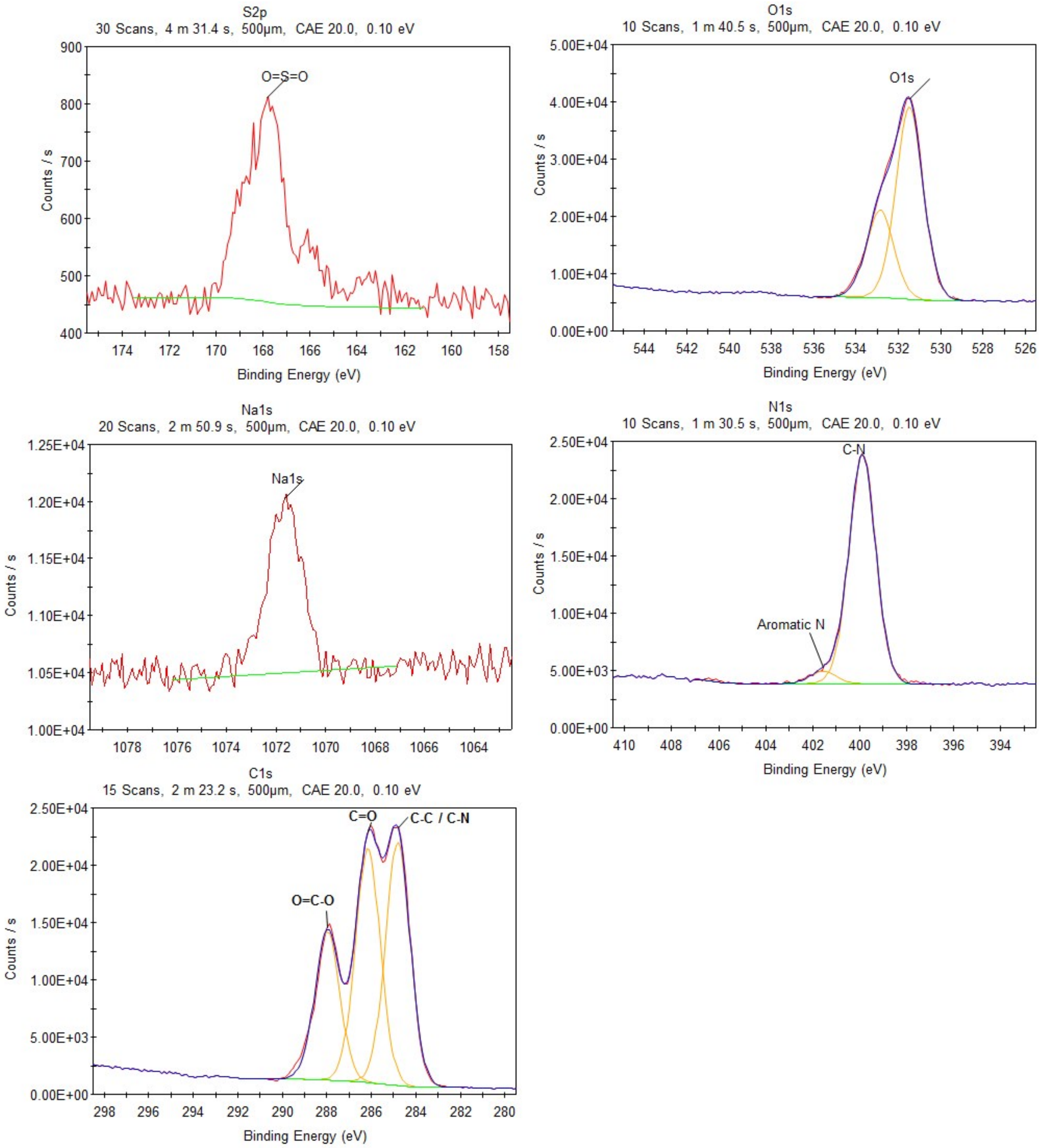


**(b) Gelatin- silver nanocomposites (G-Ag)**

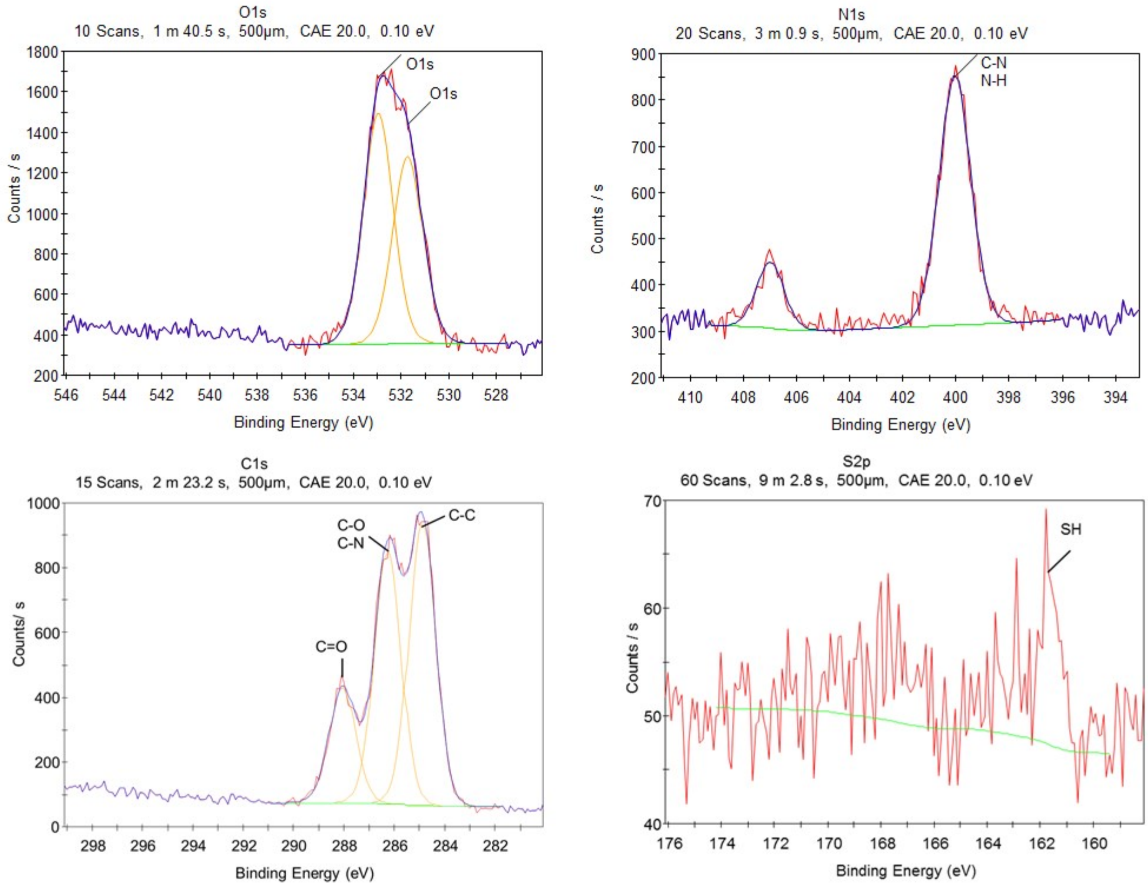


**(c) Silk fibroin- silver nanocomposites (RSF-Ag)**

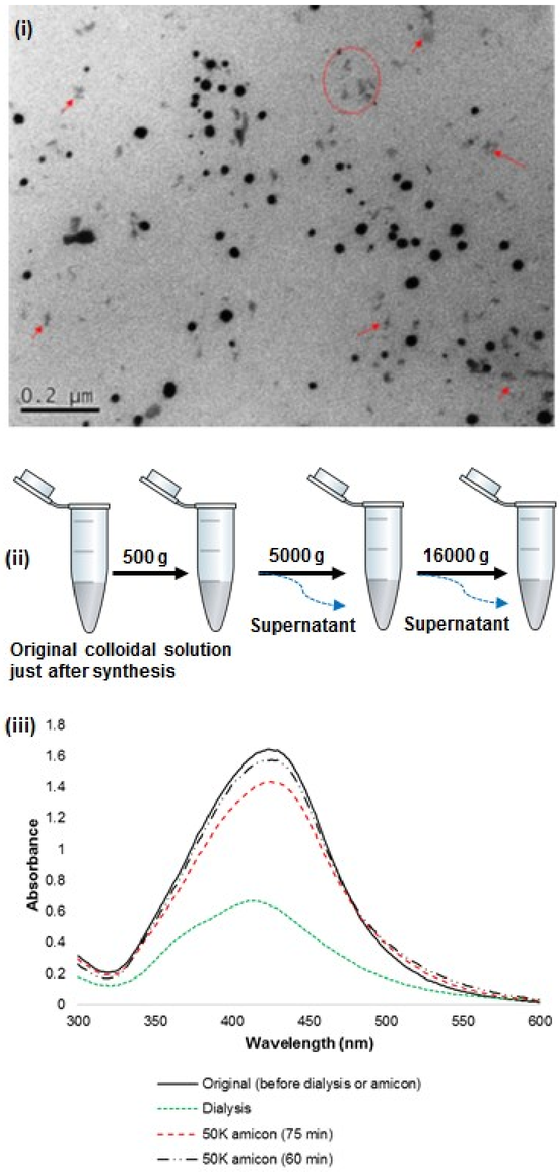


**Figure S4:** i) Representative TEM image of a nanosilver composite showing the debris associated with particles before purification; ii) Schematic of differential centrifugation protocol adopted to clean the particle preparations and obtain a largely monodisperse population of particles; iii) UV- vis spectrum indicating effects of different purification and/ or concentration techniques on the composites (spectral curves representing Amicon centrifugation were obtained after dilution of sample in 1:50).

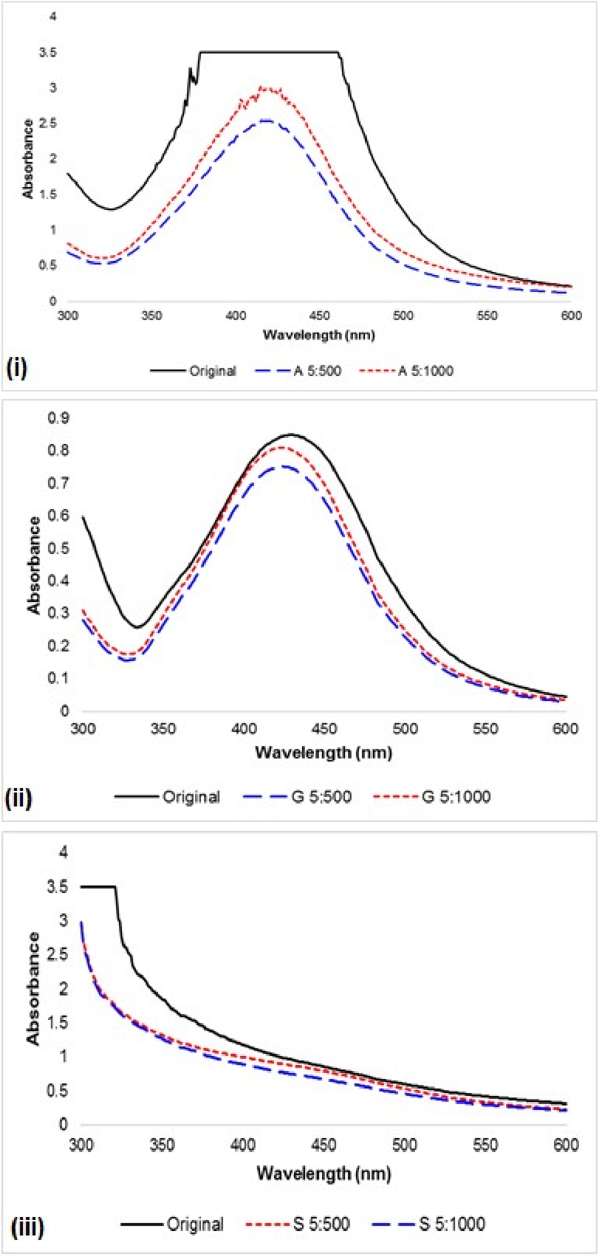


**Figure S5:** UV-visible spectra of dialyzed and undialyzed nanocomposites against two different buffer volumes with Pur-A-Lyser dialysis tubes. ; i) Alginate, ii) Gelatin, iii) Silk Fibroin

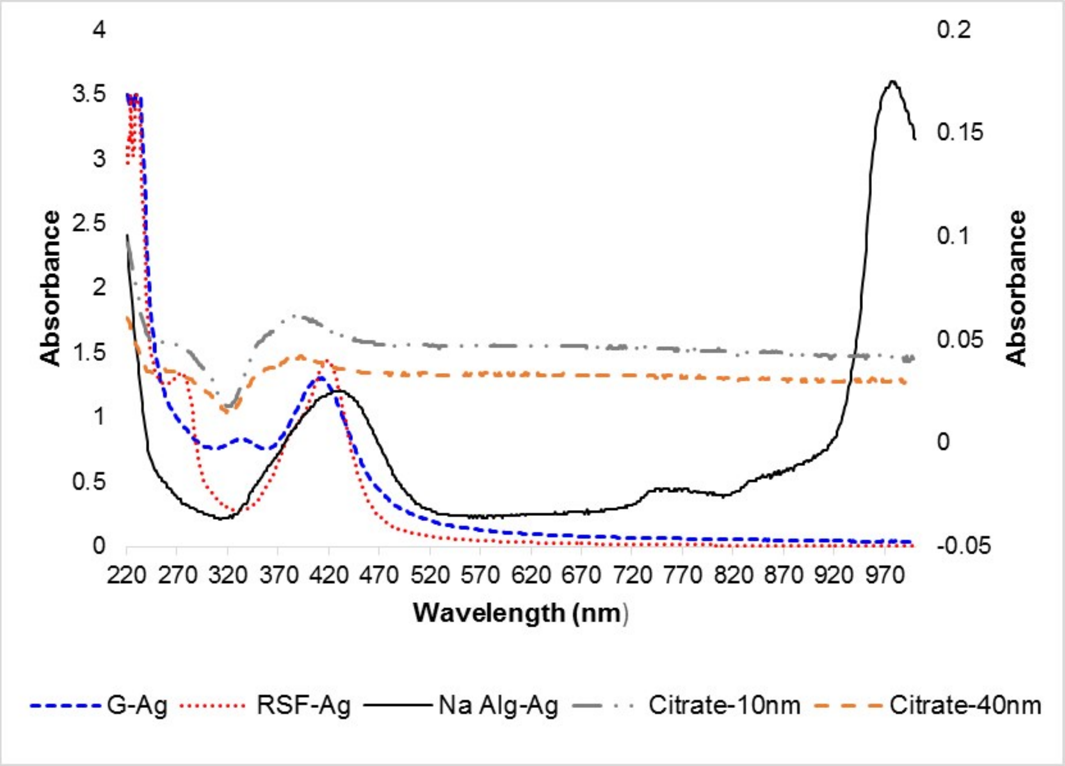


**Figure S6:** UV-vis spectral profile indicating behaviour of different nanoparticles in PBS (pH 7.4).

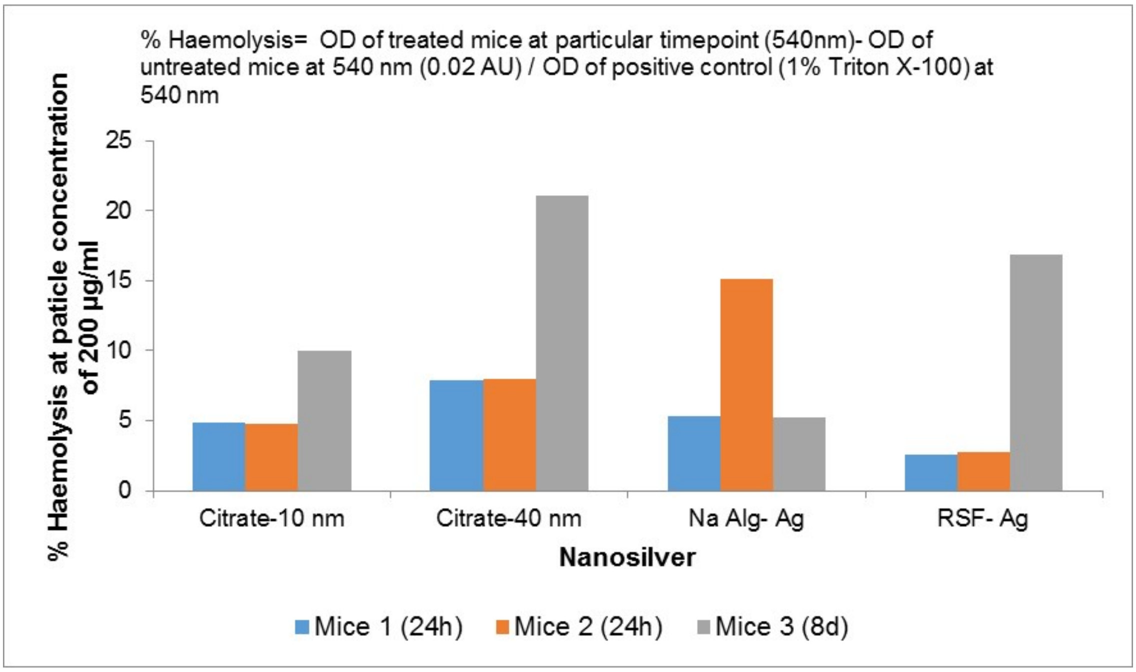


**Figure S7:** Haemolytic activity of different particles in mouse blood (blood was withdrawn from the thoracic cavity post treatment period and euthanization of animals)
