# Supplementary material for: In vitro coronal protein signatures and biological impact of silver nanoparticles synthesized with different natural polymers as capping agents
Source: Nanoscale Adv. 2021 May 17;3(15):4424–39. doi: 10.1039/d0na01013h (PMC9418127; doi:10.1039/d0na01013h)
Supplement: NA-003-D0NA01013H-s002 [file NA-003-D0NA01013H-s002.pdf]

Electronic Supplementary Material (ESI) for Nanoscale Advances.
This journal is © The Royal Society of Chemistry 2021

Na-Ag Alg

Supplementary File

Accession	Protein Name
ALBU_HUMAN	Serum albumin OS=Homo sapiens GN=ALB PE=1 SV=2
CO3_HUMAN	Complement C3 OS=Homo sapiens GN=C3 PE=1 SV=2
TRFE_HUMAN	Serotransferrin OS=Homo sapiens GN=TF PE=1 SV=3
FIBB_HUMAN	Fibrinogen beta chain OS=Homo sapiens GN=FGB PE=1 SV=2
FIBA_HUMAN	Fibrinogen alpha chain OS=Homo sapiens GN=FGA PE=1 SV=2
**APOA1_HUMAN**	**Apolipoprotein A-I OS=Homo sapiens GN=APOA1 PE=1 SV=1**
A1AT_HUMAN	Alpha-1-antitrypsin OS=Homo sapiens GN=SERPINA1 PE=1 SV=3
FIBG_HUMAN	Fibrinogen gamma chain OS=Homo sapiens GN=FGG PE=1 SV=3
HPT_HUMAN	Haptoglobin OS=Homo sapiens GN=HP PE=1 SV=1
CERU_HUMAN	Ceruloplasmin OS=Homo sapiens GN=CP PE=1 SV=1
TLN1_HUMAN	Talin-1 OS=Homo sapiens GN=TLN1 PE=1 SV=3
APOA4_HUMAN	Apolipoprotein A-IV OS=Homo sapiens GN=APOA4 PE=1 SV=3
IGHM_HUMAN	Immunoglobulin heavy constant mu OS=Homo sapiens GN=IGHM PE=1 SV=4
CO4B_HUMAN	Complement C4-B OS=Homo sapiens GN=C4B PE=1 SV=2
TRY1_HUMAN	Trypsin-1 OS=Homo sapiens GN=PRSS1 PE=1 SV=1
ITIH2_HUMAN	Inter-alpha-trypsin inhibitor heavy chain H2 OS=Homo sapiens GN=ITIH2 PE=1 SV=2
ITIH4_HUMAN	Inter-alpha-trypsin inhibitor heavy chain H4 OS=Homo sapiens GN=ITIH4 PE=1 SV=4
IGG1_HUMAN	Immunoglobulin gamma-1 heavy chain OS=Homo sapiens PE=1 SV=1
HEMO_HUMAN	Hemopexin OS=Homo sapiens GN=HPX PE=1 SV=2
FLNA_HUMAN	Filamin-A OS=Homo sapiens GN=FLNA PE=1 SV=4
FINC_HUMAN	Fibronectin OS=Homo sapiens GN=FN1 PE=1 SV=4
MYH9_HUMAN	Myosin-9 OS=Homo sapiens GN=MYH9 PE=1 SV=4
ACTB_HUMAN	Actin, cytoplasmic 1 OS=Homo sapiens GN=ACTB PE=1 SV=1
GELS_HUMAN	Gelsolin OS=Homo sapiens GN=GSN PE=1 SV=1
PLMN_HUMAN	Plasminogen OS=Homo sapiens GN=PLG PE=1 SV=2
CFAB_HUMAN	Complement factor B OS=Homo sapiens GN=CFB PE=1 SV=2
HBA_HUMAN	Hemoglobin subunit alpha OS=Homo sapiens GN=HBA1 PE=1 SV=2
KNG1_HUMAN	Kininogen-1 OS=Homo sapiens GN=KNG1 PE=1 SV=2
APOE_HUMAN	Apolipoprotein E OS=Homo sapiens GN=APOE PE=1 SV=1
HEP2_HUMAN	Heparin cofactor 2 OS=Homo sapiens GN=SERPIND1 PE=1 SV=3
HBD_HUMAN	Hemoglobin subunit delta OS=Homo sapiens GN=HBD PE=1 SV=2
VTNC_HUMAN	Vitronectin OS=Homo sapiens GN=VTN PE=1 SV=1
APOA2_HUMAN	Apolipoprotein A-II OS=Homo sapiens GN=APOA2 PE=1 SV=1
HBB_HUMAN	Hemoglobin subunit beta OS=Homo sapiens GN=HBB PE=1 SV=2

PON1_HUMAN	Serum paraoxonase/arylesterase 1 OS=Homo sapiens GN=PON1 PE=1 SV=3
FETUA_HUMAN	Alpha-2-HS-glycoprotein OS=Homo sapiens GN=AHSG PE=1 SV=1
IGHA1_HUMAN	Immunoglobulin heavy constant alpha 1 OS=Homo sapiens GN=IGHA1 PE=1 SV=2
IGHG4_HUMAN	Immunoglobulin heavy constant gamma 4 OS=Homo sapiens GN=IGHG4 PE=1 SV=1
CLUS_HUMAN	Clusterin OS=Homo sapiens GN=CLU PE=1 SV=1
IGL1_HUMAN	Immunoglobulin lambda-1 light chain OS=Homo sapiens PE=1 SV=1
IGK_HUMAN	Immunoglobulin kappa light chain OS=Homo sapiens PE=1 SV=1
HRG_HUMAN	Histidine-rich glycoprotein OS=Homo sapiens GN=HRG PE=1 SV=1
C1R_HUMAN	Complement C1r subcomponent OS=Homo sapiens GN=C1R PE=1 SV=2
A1AG2_HUMAN	Alpha-1-acid glycoprotein 2 OS=Homo sapiens GN=ORM2 PE=1 SV=2
APOH_HUMAN	Beta-2-glycoprotein 1 OS=Homo sapiens GN=APOH PE=1 SV=3
APOL1_HUMAN	Apolipoprotein L1 OS=Homo sapiens GN=APOL1 PE=1 SV=5
APOC3_HUMAN	Apolipoprotein C-III OS=Homo sapiens GN=APOC3 PE=1 SV=1
AMBP_HUMAN	Protein AMBP OS=Homo sapiens GN=AMBP PE=1 SV=1
ITA2B_HUMAN	Integrin alpha-IIb OS=Homo sapiens GN=ITGA2B PE=1 SV=3
F13A_HUMAN	Coagulation factor XIII A chain OS=Homo sapiens GN=F13A1 PE=1 SV=4
APOC2_HUMAN	Apolipoprotein C-II OS=Homo sapiens GN=APOC2 PE=1 SV=1
VINC_HUMAN	Vinculin OS=Homo sapiens GN=VCL PE=1 SV=4
PGRP2_HUMAN	N-acetylmuramoyl-L-alanine amidase OS=Homo sapiens GN=PGLYRP2 PE=1 SV=1
IGHG3_HUMAN	Immunoglobulin heavy constant gamma 3 OS=Homo sapiens GN=IGHG3 PE=1 SV=2
1433Z_HUMAN	14-3-3 protein zeta/delta OS=Homo sapiens GN=YWHAZ PE=1 SV=1
TSP1_HUMAN	Thrombospondin-1 OS=Homo sapiens GN=THBS1 PE=1 SV=2
A2AP_HUMAN	Alpha-2-antiplasmin OS=Homo sapiens GN=SERPINF2 PE=1 SV=3
ITB3_HUMAN	Integrin beta-3 OS=Homo sapiens GN=ITGB3 PE=1 SV=2
IGKC_HUMAN	Immunoglobulin kappa constant OS=Homo sapiens GN=IGKC PE=1 SV=2
TPM4_HUMAN	Tropomyosin alpha-4 chain OS=Homo sapiens GN=TPM4 PE=1 SV=3
B3AT_HUMAN	Band 3 anion transport protein OS=Homo sapiens GN=SLC4A1 PE=1 SV=3
ACTN1_HUMAN	Alpha-actinin-1 OS=Homo sapiens GN=ACTN1 PE=1 SV=2
C4BPA_HUMAN	C4b-binding protein alpha chain OS=Homo sapiens GN=C4BPA PE=1 SV=2
AFAM_HUMAN	Afamin OS=Homo sapiens GN=AFM PE=1 SV=1
IGHG2_HUMAN	Immunoglobulin heavy constant gamma 2 OS=Homo sapiens GN=IGHG2 PE=1 SV=2
FCN3_HUMAN	Ficolin-3 OS=Homo sapiens GN=FCN3 PE=1 SV=2
PROS_HUMAN	Vitamin K-dependent protein S OS=Homo sapiens GN=PROS1 PE=1 SV=1
APOC1_HUMAN	Apolipoprotein C-I OS=Homo sapiens GN=APOC1 PE=1 SV=1
IGLC2_HUMAN	Immunoglobulin lambda constant 2 OS=Homo sapiens GN=IGLC2 PE=1 SV=1
HPTR_HUMAN	Haptoglobin-related protein OS=Homo sapiens GN=HPR PE=2 SV=2
A1AG1_HUMAN	Alpha-1-acid glycoprotein 1 OS=Homo sapiens GN=ORM1 PE=1 SV=1

C1QB_HUMAN	Complement C1q subcomponent subunit B OS=Homo sapiens GN=C1QB PE=1 SV=3
URP2_HUMAN	Fermitin family homolog 3 OS=Homo sapiens GN=FERMT3 PE=1 SV=1
APOA_HUMAN	Apolipoprotein(a) OS=Homo sapiens GN=LPA PE=1 SV=1
TBB1_HUMAN	Tubulin beta-1 chain OS=Homo sapiens GN=TUBB1 PE=1 SV=1
PRDX2_HUMAN	Peroxiredoxin-2 OS=Homo sapiens GN=PRDX2 PE=1 SV=5
RAP1B_HUMAN	Ras-related protein Rap-1b OS=Homo sapiens GN=RAP1B PE=1 SV=1
CD5L_HUMAN	CD5 antigen-like OS=Homo sapiens GN=CD5L PE=1 SV=1
TBA4A_HUMAN	Tubulin alpha-4A chain OS=Homo sapiens GN=TUBA4A PE=1 SV=1
SPTB1_HUMAN	**Spectrin beta chain, erythrocytic OS=Homo sapiens GN=SPTB PE=1 SV=5**
C1QA_HUMAN	Complement C1q subcomponent subunit A OS=Homo sapiens GN=C1QA PE=1 SV=2
G3P_HUMAN	Glyceraldehyde-3-phosphate dehydrogenase OS=Homo sapiens GN=GAPDH PE=1 SV=3
TAGL2_HUMAN	Transgelin-2 OS=Homo sapiens GN=TAGLN2 PE=1 SV=3
SPTA1_HUMAN	**Spectrin alpha chain, erythrocytic 1 OS=Homo sapiens GN=SPTA1 PE=1 SV=5**
ZPI_HUMAN	Protein Z-dependent protease inhibitor OS=Homo sapiens GN=SERPINA10 PE=1 SV=1
PROF1_HUMAN	Profilin-1 OS=Homo sapiens GN=PFN1 PE=1 SV=2
IGA2_HUMAN	Immunoglobulin alpha-2 heavy chain OS=Homo sapiens PE=1 SV=1
STOM_HUMAN	Erythrocyte band 7 integral membrane protein OS=Homo sapiens GN=STOM PE=1 SV=3
IGJ_HUMAN	Immunoglobulin J chain OS=Homo sapiens GN=JCHAIN PE=1 SV=4
COF1_HUMAN	Cofilin-1 OS=Homo sapiens GN=CFL1 PE=1 SV=3
C1QC_HUMAN	Complement C1q subcomponent subunit C OS=Homo sapiens GN=C1QC PE=1 SV=3
ENOA_HUMAN	Alpha-enolase OS=Homo sapiens GN=ENO1 PE=1 SV=2
MYL6_HUMAN	Myosin light polypeptide 6 OS=Homo sapiens GN=MYL6 PE=1 SV=2
KPYM_HUMAN	Pyruvate kinase PKM OS=Homo sapiens GN=PKM PE=1 SV=4
CXCL7_HUMAN	Platelet basic protein OS=Homo sapiens GN=PPBP PE=1 SV=3
SAA4_HUMAN	Serum amyloid A-4 protein OS=Homo sapiens GN=SAA4 PE=1 SV=2
KV320_HUMAN	Immunoglobulin kappa variable 3-20 OS=Homo sapiens GN=IGKV3-20 PE=1 SV=2
S10A8_HUMAN	Protein S100-A8 OS=Homo sapiens GN=S100A8 PE=1 SV=1
ML12A_HUMAN	Myosin regulatory light chain 12A OS=Homo sapiens GN=MYL12A PE=1 SV=2
HABP2_HUMAN	Hyaluronan-binding protein 2 OS=Homo sapiens GN=HABP2 PE=1 SV=1
PRG4_HUMAN	Proteoglycan 4 OS=Homo sapiens GN=PRG4 PE=1 SV=2
MOES_HUMAN	Moesin OS=Homo sapiens GN=MSN PE=1 SV=3
CAVN2_HUMAN	Caveolae-associated protein 2 OS=Homo sapiens GN=CAVIN2 PE=1 SV=3
CAP1_HUMAN	Adenylyl cyclase-associated protein 1 OS=Homo sapiens GN=CAP1 PE=1 SV=5
GP1BA_HUMAN	Platelet glycoprotein Ib alpha chain OS=Homo sapiens GN=GP1BA PE=1 SV=2
ILK_HUMAN	Integrin-linked protein kinase OS=Homo sapiens GN=ILK PE=1 SV=2
LDHB_HUMAN	L-lactate dehydrogenase B chain OS=Homo sapiens GN=LDHB PE=1 SV=2
ALDOA_HUMAN	Fructose-bisphosphate aldolase A OS=Homo sapiens GN=ALDOA PE=1 SV=2

PLEK_HUMAN	Pleckstrin OS=Homo sapiens GN=PLEK PE=1 SV=3
PLF4_HUMAN	Platelet factor 4 OS=Homo sapiens GN=PF4 PE=1 SV=2
PPIA_HUMAN	Peptidyl-prolyl cis-trans isomerase A OS=Homo sapiens GN=PPIA PE=1 SV=2
HSP7C_HUMAN	Heat shock cognate 71 kDa protein OS=Homo sapiens GN=HSPA8 PE=1 SV=1
GTR14_HUMAN	Solute carrier family 2, facilitated glucose transporter member 14 OS=Homo sapiens GN=SLC2A14 PE=2 SV=1
HV374_HUMAN	Immunoglobulin heavy variable 3-74 OS=Homo sapiens GN=IGHV3-74 PE=3 SV=1
COR1A_HUMAN	Coronin-1A OS=Homo sapiens GN=CORO1A PE=1 SV=4
TBB5_HUMAN	Tubulin beta chain OS=Homo sapiens GN=TUBB PE=1 SV=2
RSU1_HUMAN	Ras suppressor protein 1 OS=Homo sapiens GN=RSU1 PE=1 SV=3
CATA_HUMAN	Catalase OS=Homo sapiens GN=CAT PE=1 SV=3
TYB4_HUMAN	Thymosin beta-4 OS=Homo sapiens GN=TMSB4X PE=1 SV=2
GP1BB_HUMAN	Platelet glycoprotein Ib beta chain OS=Homo sapiens GN=GP1BB PE=1 SV=1
KV401_HUMAN	Immunoglobulin kappa variable 4-1 OS=Homo sapiens GN=IGKV4-1 PE=1 SV=1
H2B1B_HUMAN	Histone H2B type 1-B OS=Homo sapiens GN=HIST1H2BB PE=1 SV=2
1433E_HUMAN	14-3-3 protein epsilon OS=Homo sapiens GN=YWHAE PE=1 SV=1
1B35_HUMAN	HLA class I histocompatibility antigen, B-35 alpha chain OS=Homo sapiens GN=HLA-B PE=1 SV=1
CAH1_HUMAN	Carbonic anhydrase 1 OS=Homo sapiens GN=CA1 PE=1 SV=2
WDR1_HUMAN	WD repeat-containing protein 1 OS=Homo sapiens GN=WDR1 PE=1 SV=4
KV230_HUMAN	Immunoglobulin kappa variable 2-30 OS=Homo sapiens GN=IGKV2-30 PE=3 SV=2
KV315_HUMAN	Immunoglobulin kappa variable 3-15 OS=Homo sapiens GN=IGKV3-15 PE=1 SV=2
ANK1_HUMAN	Ankyrin-1 OS=Homo sapiens GN=ANK1 PE=1 SV=3
CRAC1_HUMAN	Cartilage acidic protein 1 OS=Homo sapiens GN=CRTAC1 PE=1 SV=2
GSTO1_HUMAN	Glutathione S-transferase omega-1 OS=Homo sapiens GN=GSTO1 PE=1 SV=2
BIN2_HUMAN	Bridging integrator 2 OS=Homo sapiens GN=BIN2 PE=1 SV=3
HV434_HUMAN	Immunoglobulin heavy variable 4-34 OS=Homo sapiens GN=IGHV4-34 PE=1 SV=2
PDIA3_HUMAN	Protein disulfide-isomerase A3 OS=Homo sapiens GN=PDIA3 PE=1 SV=4
B2MG_HUMAN	Beta-2-microglobulin OS=Homo sapiens GN=B2M PE=1 SV=1
TPIS_HUMAN	Triosephosphate isomerase OS=Homo sapiens GN=TPI1 PE=1 SV=3
GTR1_HUMAN	Solute carrier family 2, facilitated glucose transporter member 1 OS=Homo sapiens GN=SLC2A1 PE=1 SV=2
GPIX_HUMAN	Platelet glycoprotein IX OS=Homo sapiens GN=GP9 PE=1 SV=3
TERA_HUMAN	Transitional endoplasmic reticulum ATPase OS=Homo sapiens GN=VCP PE=1 SV=4
ZYX_HUMAN	Zyxin OS=Homo sapiens GN=ZYX PE=1 SV=1
PGK1_HUMAN	Phosphoglycerate kinase 1 OS=Homo sapiens GN=PGK1 PE=1 SV=3
PRDX6_HUMAN	Peroxiredoxin-6 OS=Homo sapiens GN=PRDX6 PE=1 SV=3
RAB1B_HUMAN	Ras-related protein Rab-1B OS=Homo sapiens GN=RAB1B PE=1 SV=1
PNPH_HUMAN	Purine nucleoside phosphorylase OS=Homo sapiens GN=PNP PE=1 SV=2
VASP_HUMAN	Vasodilator-stimulated phosphoprotein OS=Homo sapiens GN=VASP PE=1 SV=3

1433G_HUMAN	14-3-3 protein gamma OS=Homo sapiens GN=YWHAG PE=1 SV=2
CALR_HUMAN	Calreticulin OS=Homo sapiens GN=CALR PE=1 SV=1
TRFL_HUMAN	Lactotransferrin OS=Homo sapiens GN=LTF PE=1 SV=6
COR1C_HUMAN	Coronin-1C OS=Homo sapiens GN=CORO1C PE=1 SV=1
K2C1_HUMAN	Keratin, type II cytoskeletal 1 OS=Homo sapiens GN=KRT1 PE=1 SV=6
ARF1_HUMAN	ADP-ribosylation factor 1 OS=Homo sapiens GN=ARF1 PE=1 SV=2
CAZA1_HUMAN	F-actin-capping protein subunit alpha-1 OS=Homo sapiens GN=CAPZA1 PE=1 SV=3
PDLI1_HUMAN	PDZ and LIM domain protein 1 OS=Homo sapiens GN=PDLIM1 PE=1 SV=4
EF1A1_HUMAN	Elongation factor 1-alpha 1 OS=Homo sapiens GN=EEF1A1 PE=1 SV=1
RB11A_HUMAN	Ras-related protein Rab-11A OS=Homo sapiens GN=RAB11A PE=1 SV=3
K1C10_HUMAN	Keratin, type I cytoskeletal 10 OS=Homo sapiens GN=KRT10 PE=1 SV=6
LDHA_HUMAN	L-lactate dehydrogenase A chain OS=Homo sapiens GN=LDHA PE=1 SV=2
GBB1_HUMAN	Guanine nucleotide-binding protein G(I)/G(S)/G(T) subunit beta-1 OS=Homo sapiens GN=GNB1 PE=1 SV=3
TBA1B_HUMAN	Tubulin alpha-1B chain OS=Homo sapiens GN=TUBA1B PE=1 SV=1
CALM1_HUMAN	Calmodulin-1 OS=Homo sapiens GN=CALM1 PE=1 SV=1
LEGL_HUMAN	Galectin-related protein OS=Homo sapiens GN=LGALSL PE=1 SV=2
H4_HUMAN	Histone H4 OS=Homo sapiens GN=HIST1H4A PE=1 SV=2
CAN1_HUMAN	Calpain-1 catalytic subunit OS=Homo sapiens GN=CAPN1 PE=1 SV=1
BLVRB_HUMAN	Flavin reductase (NADPH) OS=Homo sapiens GN=BLVRB PE=1 SV=3
HEM2_HUMAN	Delta-aminolevulinic acid dehydratase OS=Homo sapiens GN=ALAD PE=1 SV=1
ARPC4_HUMAN	Actin-related protein 2/3 complex subunit 4 OS=Homo sapiens GN=ARPC4 PE=1 SV=3

G- AgNP

**Accession**	**Protein Name**
ALBU_HUMAN	Serum albumin OS=Homo sapiens GN=ALB PE=1 SV=2
CO3_HUMAN	Complement C3 OS=Homo sapiens GN=C3 PE=1 SV=2
TRFE_HUMAN	Serotransferrin OS=Homo sapiens GN=TF PE=1 SV=3
FIBB_HUMAN	Fibrinogen beta chain OS=Homo sapiens GN=FGB PE=1 SV=2
A2MG_HUMAN	Alpha-2-macroglobulin OS=Homo sapiens GN=A2M PE=1 SV=3
FIBA_HUMAN	Fibrinogen alpha chain OS=Homo sapiens GN=FGA PE=1 SV=2
**APOA1_HUMAN**	**Apolipoprotein A-I OS=Homo sapiens GN=APOA1 PE=1 SV=1**
A1AT_HUMAN	Alpha-1-antitrypsin OS=Homo sapiens GN=SERPINA1 PE=1 SV=3
FIBG_HUMAN	Fibrinogen gamma chain OS=Homo sapiens GN=FGG PE=1 SV=3
HPT_HUMAN	Haptoglobin OS=Homo sapiens GN=HP PE=1 SV=1
CERU_HUMAN	Ceruloplasmin OS=Homo sapiens GN=CP PE=1 SV=1
TLN1_HUMAN	Talin-1 OS=Homo sapiens GN=TLN1 PE=1 SV=3

APOA4_HUMAN	Apolipoprotein A-IV OS=Homo sapiens GN=APOA4 PE=1 SV=3
CFAH_HUMAN	Complement factor H OS=Homo sapiens GN=CFH PE=1 SV=4
IGHM_HUMAN	Immunoglobulin heavy constant mu OS=Homo sapiens GN=IGHM PE=1 SV=4
CO4B_HUMAN	Complement C4-B OS=Homo sapiens GN=C4B PE=1 SV=2
TRY1_HUMAN	Trypsin-1 OS=Homo sapiens GN=PRSS1 PE=1 SV=1
ITIH2_HUMAN	Inter-alpha-trypsin inhibitor heavy chain H2 OS=Homo sapiens GN=ITIH2 PE=1 SV=2
ITIH4_HUMAN	Inter-alpha-trypsin inhibitor heavy chain H4 OS=Homo sapiens GN=ITIH4 PE=1 SV=4
IGG1_HUMAN	Immunoglobulin gamma-1 heavy chain OS=Homo sapiens PE=1 SV=1
HEMO_HUMAN	Hemopexin OS=Homo sapiens GN=HPX PE=1 SV=2
FLNA_HUMAN	Filamin-A OS=Homo sapiens GN=FLNA PE=1 SV=4
ANT3_HUMAN	Antithrombin-III OS=Homo sapiens GN=SERPINC1 PE=1 SV=1
FINC_HUMAN	Fibronectin OS=Homo sapiens GN=FN1 PE=1 SV=4
MYH9_HUMAN	Myosin-9 OS=Homo sapiens GN=MYH9 PE=1 SV=4
ACTB_HUMAN	Actin, cytoplasmic 1 OS=Homo sapiens GN=ACTB PE=1 SV=1
GELS_HUMAN	Gelsolin OS=Homo sapiens GN=GSN PE=1 SV=1
PLMN_HUMAN	Plasminogen OS=Homo sapiens GN=PLG PE=1 SV=2
HBA_HUMAN	Hemoglobin subunit alpha OS=Homo sapiens GN=HBA1 PE=1 SV=2
KNG1_HUMAN	Kininogen-1 OS=Homo sapiens GN=KNG1 PE=1 SV=2
APOE_HUMAN	Apolipoprotein E OS=Homo sapiens GN=APOE PE=1 SV=1
HEP2_HUMAN	Heparin cofactor 2 OS=Homo sapiens GN=SERPIND1 PE=1 SV=3
HBD_HUMAN	Hemoglobin subunit delta OS=Homo sapiens GN=HBD PE=1 SV=2
VTNC_HUMAN	Vitronectin OS=Homo sapiens GN=VTN PE=1 SV=1
APOA2_HUMAN	Apolipoprotein A-II OS=Homo sapiens GN=APOA2 PE=1 SV=1
HBB_HUMAN	Hemoglobin subunit beta OS=Homo sapiens GN=HBB PE=1 SV=2
PON1_HUMAN	Serum paraoxonase/arylesterase 1 OS=Homo sapiens GN=PON1 PE=1 SV=3
FETUA_HUMAN	Alpha-2-HS-glycoprotein OS=Homo sapiens GN=AHSG PE=1 SV=1
AACT_HUMAN	Alpha-1-antichymotrypsin OS=Homo sapiens GN=SERPINA3 PE=1 SV=2
IGHA1_HUMAN	Immunoglobulin heavy constant alpha 1 OS=Homo sapiens GN=IGHA1 PE=1 SV=2
IGHG4_HUMAN	Immunoglobulin heavy constant gamma 4 OS=Homo sapiens GN=IGHG4 PE=1 SV=1
CLUS_HUMAN	Clusterin OS=Homo sapiens GN=CLU PE=1 SV=1
IGL1_HUMAN	Immunoglobulin lambda-1 light chain OS=Homo sapiens PE=1 SV=1
IGK_HUMAN	Immunoglobulin kappa light chain OS=Homo sapiens PE=1 SV=1
A1BG_HUMAN	Alpha-1B-glycoprotein OS=Homo sapiens GN=A1BG PE=1 SV=4
HRG_HUMAN	Histidine-rich glycoprotein OS=Homo sapiens GN=HRG PE=1 SV=1
C1R_HUMAN	Complement C1r subcomponent OS=Homo sapiens GN=C1R PE=1 SV=2
A1AG2_HUMAN	Alpha-1-acid glycoprotein 2 OS=Homo sapiens GN=ORM2 PE=1 SV=2
APOH_HUMAN	Beta-2-glycoprotein 1 OS=Homo sapiens GN=APOH PE=1 SV=3

KLKB1_HUMAN	Plasma kallikrein OS=Homo sapiens GN=KLKB1 PE=1 SV=1
APOL1_HUMAN	Apolipoprotein L1 OS=Homo sapiens GN=APOL1 PE=1 SV=5
APOC3_HUMAN	Apolipoprotein C-III OS=Homo sapiens GN=APOC3 PE=1 SV=1
AMBP_HUMAN	Protein AMBP OS=Homo sapiens GN=AMBP PE=1 SV=1
ITA2B_HUMAN	Integrin alpha-IIb OS=Homo sapiens GN=ITGA2B PE=1 SV=3
F13A_HUMAN	Coagulation factor XIII A chain OS=Homo sapiens GN=F13A1 PE=1 SV=4
APOC2_HUMAN	Apolipoprotein C-II OS=Homo sapiens GN=APOC2 PE=1 SV=1
VINC_HUMAN	Vinculin OS=Homo sapiens GN=VCL PE=1 SV=4
PGRP2_HUMAN	N-acetylmuramoyl-L-alanine amidase OS=Homo sapiens GN=PGLYRP2 PE=1 SV=1
IGHG3_HUMAN	Immunoglobulin heavy constant gamma 3 OS=Homo sapiens GN=IGHG3 PE=1 SV=2
1433Z_HUMAN	14-3-3 protein zeta/delta OS=Homo sapiens GN=YWHAZ PE=1 SV=1
TSP1_HUMAN	Thrombospondin-1 OS=Homo sapiens GN=THBS1 PE=1 SV=2
A2AP_HUMAN	Alpha-2-antiplasmin OS=Homo sapiens GN=SERPINF2 PE=1 SV=3
ITB3_HUMAN	Integrin beta-3 OS=Homo sapiens GN=ITGB3 PE=1 SV=2
PEDF_HUMAN	Pigment epithelium-derived factor OS=Homo sapiens GN=SERPINF1 PE=1 SV=4
IGKC_HUMAN	Immunoglobulin kappa constant OS=Homo sapiens GN=IGKC PE=1 SV=2
TPM4_HUMAN	Tropomyosin alpha-4 chain OS=Homo sapiens GN=TPM4 PE=1 SV=3
B3AT_HUMAN	Band 3 anion transport protein OS=Homo sapiens GN=SLC4A1 PE=1 SV=3
ACTN1_HUMAN	Alpha-actinin-1 OS=Homo sapiens GN=ACTN1 PE=1 SV=2
AFAM_HUMAN	Afamin OS=Homo sapiens GN=AFM PE=1 SV=1
TTHY_HUMAN	Transthyretin OS=Homo sapiens GN=TTR PE=1 SV=1
CO9_HUMAN	Complement component C9 OS=Homo sapiens GN=C9 PE=1 SV=2
IGHG2_HUMAN	Immunoglobulin heavy constant gamma 2 OS=Homo sapiens GN=IGHG2 PE=1 SV=2
FCN3_HUMAN	Ficolin-3 OS=Homo sapiens GN=FCN3 PE=1 SV=2
PROS_HUMAN	Vitamin K-dependent protein S OS=Homo sapiens GN=PROS1 PE=1 SV=1
APOC1_HUMAN	Apolipoprotein C-I OS=Homo sapiens GN=APOC1 PE=1 SV=1
IGLC2_HUMAN	Immunoglobulin lambda constant 2 OS=Homo sapiens GN=IGLC2 PE=1 SV=1
HPTR_HUMAN	Haptoglobin-related protein OS=Homo sapiens GN=HPR PE=2 SV=2
CO1A1_HUMAN	Collagen alpha-1(I) chain OS=Homo sapiens GN=COL1A1 PE=1 SV=5
C1QB_HUMAN	Complement C1q subcomponent subunit B OS=Homo sapiens GN=C1QB PE=1 SV=3
URP2_HUMAN	Fermitin family homolog 3 OS=Homo sapiens GN=FERMT3 PE=1 SV=1
APOA_HUMAN	Apolipoprotein(a) OS=Homo sapiens GN=LPA PE=1 SV=1
TBB1_HUMAN	Tubulin beta-1 chain OS=Homo sapiens GN=TUBB1 PE=1 SV=1
PRDX2_HUMAN	Peroxiredoxin-2 OS=Homo sapiens GN=PRDX2 PE=1 SV=5
RAP1B_HUMAN	Ras-related protein Rap-1b OS=Homo sapiens GN=RAP1B PE=1 SV=1
CD5L_HUMAN	CD5 antigen-like OS=Homo sapiens GN=CD5L PE=1 SV=1
TBA4A_HUMAN	Tubulin alpha-4A chain OS=Homo sapiens GN=TUBA4A PE=1 SV=1

**SPTB1_HUMAN**	**Spectrin beta chain, erythrocytic OS=Homo sapiens GN=SPTB PE=1 SV=5**
C1QA_HUMAN	Complement C1q subcomponent subunit A OS=Homo sapiens GN=C1QA PE=1 SV=2
G3P_HUMAN	Glyceraldehyde-3-phosphate dehydrogenase OS=Homo sapiens GN=GAPDH PE=1 SV=3
TAGL2_HUMAN	Transgelin-2 OS=Homo sapiens GN=TAGLN2 PE=1 SV=3
**SPTA1_HUMAN**	**Spectrin alpha chain, erythrocytic 1 OS=Homo sapiens GN=SPTA1 PE=1 SV=5**
ZPI_HUMAN	Protein Z-dependent protease inhibitor OS=Homo sapiens GN=SERPINA10 PE=1 SV=1
PROF1_HUMAN	Profilin-1 OS=Homo sapiens GN=PFN1 PE=1 SV=2
CO1A2_HUMAN	Collagen alpha-2(I) chain OS=Homo sapiens GN=COL1A2 PE=1 SV=7
IGA2_HUMAN	Immunoglobulin alpha-2 heavy chain OS=Homo sapiens PE=1 SV=1
S10A9_HUMAN	Protein S100-A9 OS=Homo sapiens GN=S100A9 PE=1 SV=1
STOM_HUMAN	Erythrocyte band 7 integral membrane protein OS=Homo sapiens GN=STOM PE=1 SV=3
LBP_HUMAN	Lipopolysaccharide-binding protein OS=Homo sapiens GN=LBP PE=1 SV=3
COF1_HUMAN	Cofilin-1 OS=Homo sapiens GN=CFL1 PE=1 SV=3
ENOA_HUMAN	Alpha-enolase OS=Homo sapiens GN=ENO1 PE=1 SV=2
MYL6_HUMAN	Myosin light polypeptide 6 OS=Homo sapiens GN=MYL6 PE=1 SV=2
KPYM_HUMAN	Pyruvate kinase PKM OS=Homo sapiens GN=PKM PE=1 SV=4
CXCL7_HUMAN	Platelet basic protein OS=Homo sapiens GN=PPBP PE=1 SV=3
KV320_HUMAN	Immunoglobulin kappa variable 3-20 OS=Homo sapiens GN=IGKV3-20 PE=1 SV=2
S10A8_HUMAN	Protein S100-A8 OS=Homo sapiens GN=S100A8 PE=1 SV=1
ML12A_HUMAN	Myosin regulatory light chain 12A OS=Homo sapiens GN=MYL12A PE=1 SV=2
HABP2_HUMAN	Hyaluronan-binding protein 2 OS=Homo sapiens GN=HABP2 PE=1 SV=1
MOES_HUMAN	Moesin OS=Homo sapiens GN=MSN PE=1 SV=3
CAVN2_HUMAN	Caveolae-associated protein 2 OS=Homo sapiens GN=CAVIN2 PE=1 SV=3
CAP1_HUMAN	Adenylyl cyclase-associated protein 1 OS=Homo sapiens GN=CAP1 PE=1 SV=5
GP1BA_HUMAN	Platelet glycoprotein Ib alpha chain OS=Homo sapiens GN=GP1BA PE=1 SV=2
ILK_HUMAN	Integrin-linked protein kinase OS=Homo sapiens GN=ILK PE=1 SV=2
LDHB_HUMAN	L-lactate dehydrogenase B chain OS=Homo sapiens GN=LDHB PE=1 SV=2
ALDOA_HUMAN	Fructose-bisphosphate aldolase A OS=Homo sapiens GN=ALDOA PE=1 SV=2
PLEK_HUMAN	Pleckstrin OS=Homo sapiens GN=PLEK PE=1 SV=3
CO4A_HUMAN	Complement C4-A OS=Homo sapiens GN=C4A PE=1 SV=2
PLF4_HUMAN	Platelet factor 4 OS=Homo sapiens GN=PF4 PE=1 SV=2
PPIA_HUMAN	Peptidyl-prolyl cis-trans isomerase A OS=Homo sapiens GN=PPIA PE=1 SV=2
HSP7C_HUMAN	Heat shock cognate 71 kDa protein OS=Homo sapiens GN=HSPA8 PE=1 SV=1
GTR14_HUMAN	Solute carrier family 2, facilitated glucose transporter member 14 OS=Homo sapiens GN=SLC2A14 PE=2 SV=1
COR1A_HUMAN	Coronin-1A OS=Homo sapiens GN=CORO1A PE=1 SV=4
TBB5_HUMAN	Tubulin beta chain OS=Homo sapiens GN=TUBB PE=1 SV=2
RSU1_HUMAN	Ras suppressor protein 1 OS=Homo sapiens GN=RSU1 PE=1 SV=3

CATA_HUMAN	Catalase OS=Homo sapiens GN=CAT PE=1 SV=3
TYB4_HUMAN	Thymosin beta-4 OS=Homo sapiens GN=TMSB4X PE=1 SV=2
GP1BB_HUMAN	Platelet glycoprotein Ib beta chain OS=Homo sapiens GN=GP1BB PE=1 SV=1
KV401_HUMAN	Immunoglobulin kappa variable 4-1 OS=Homo sapiens GN=IGKV4-1 PE=1 SV=1
H2B1B_HUMAN	Histone H2B type 1-B OS=Homo sapiens GN=HIST1H2BB PE=1 SV=2
1433E_HUMAN	14-3-3 protein epsilon OS=Homo sapiens GN=YWHAE PE=1 SV=1
1B35_HUMAN	HLA class I histocompatibility antigen, B-35 alpha chain OS=Homo sapiens GN=HLA-B PE=1 SV=1
CAH1_HUMAN	Carbonic anhydrase 1 OS=Homo sapiens GN=CA1 PE=1 SV=2
WDR1_HUMAN	WD repeat-containing protein 1 OS=Homo sapiens GN=WDR1 PE=1 SV=4
KV230_HUMAN	Immunoglobulin kappa variable 2-30 OS=Homo sapiens GN=IGKV2-30 PE=3 SV=2
ANK1_HUMAN	Ankyrin-1 OS=Homo sapiens GN=ANK1 PE=1 SV=3
KV311_HUMAN	Immunoglobulin kappa variable 3-11 OS=Homo sapiens GN=IGKV3-11 PE=1 SV=1
CRAC1_HUMAN	Cartilage acidic protein 1 OS=Homo sapiens GN=CRTAC1 PE=1 SV=2
GSTO1_HUMAN	Glutathione S-transferase omega-1 OS=Homo sapiens GN=GSTO1 PE=1 SV=2
BIN2_HUMAN	Bridging integrator 2 OS=Homo sapiens GN=BIN2 PE=1 SV=3
HV434_HUMAN	Immunoglobulin heavy variable 4-34 OS=Homo sapiens GN=IGHV4-34 PE=1 SV=2
PDIA3_HUMAN	Protein disulfide-isomerase A3 OS=Homo sapiens GN=PDIA3 PE=1 SV=4
B2MG_HUMAN	Beta-2-microglobulin OS=Homo sapiens GN=B2M PE=1 SV=1
TPIS_HUMAN	Triosephosphate isomerase OS=Homo sapiens GN=TPI1 PE=1 SV=3
GTR1_HUMAN	Solute carrier family 2, facilitated glucose transporter member 1 OS=Homo sapiens GN=SLC2A1 PE=1 SV=2
GPIX_HUMAN	Platelet glycoprotein IX OS=Homo sapiens GN=GP9 PE=1 SV=3
TERA_HUMAN	Transitional endoplasmic reticulum ATPase OS=Homo sapiens GN=VCP PE=1 SV=4
ZYX_HUMAN	Zyxin OS=Homo sapiens GN=ZYX PE=1 SV=1
PGK1_HUMAN	Phosphoglycerate kinase 1 OS=Homo sapiens GN=PGK1 PE=1 SV=3
GPV_HUMAN	Platelet glycoprotein V OS=Homo sapiens GN=GP5 PE=1 SV=1
PRDX6_HUMAN	Peroxiredoxin-6 OS=Homo sapiens GN=PRDX6 PE=1 SV=3
RAB1B_HUMAN	Ras-related protein Rab-1B OS=Homo sapiens GN=RAB1B PE=1 SV=1
PNPH_HUMAN	Purine nucleoside phosphorylase OS=Homo sapiens GN=PNP PE=1 SV=2
VASP_HUMAN	Vasodilator-stimulated phosphoprotein OS=Homo sapiens GN=VASP PE=1 SV=3
1433G_HUMAN	14-3-3 protein gamma OS=Homo sapiens GN=YWHAG PE=1 SV=2
CALR_HUMAN	Calreticulin OS=Homo sapiens GN=CALR PE=1 SV=1
CO3A1_HUMAN	Collagen alpha-1(III) chain OS=Homo sapiens GN=COL3A1 PE=1 SV=4
TRFL_HUMAN	Lactotransferrin OS=Homo sapiens GN=LTF PE=1 SV=6
COR1C_HUMAN	Coronin-1C OS=Homo sapiens GN=CORO1C PE=1 SV=1
ARF1_HUMAN	ADP-ribosylation factor 1 OS=Homo sapiens GN=ARF1 PE=1 SV=2
RET4_HUMAN	Retinol-binding protein 4 OS=Homo sapiens GN=RBP4 PE=1 SV=3
CAZA1_HUMAN	F-actin-capping protein subunit alpha-1 OS=Homo sapiens GN=CAPZA1 PE=1 SV=3

PDLI1_HUMAN	PDZ and LIM domain protein 1 OS=Homo sapiens GN=PDLIM1 PE=1 SV=4
EF1A1_HUMAN	Elongation factor 1-alpha 1 OS=Homo sapiens GN=EEF1A1 PE=1 SV=1
RB11A_HUMAN	Ras-related protein Rab-11A OS=Homo sapiens GN=RAB11A PE=1 SV=3
LDHA_HUMAN	L-lactate dehydrogenase A chain OS=Homo sapiens GN=LDHA PE=1 SV=2
TBA1B_HUMAN	Tubulin alpha-1B chain OS=Homo sapiens GN=TUBA1B PE=1 SV=1
CALM1_HUMAN	Calmodulin-1 OS=Homo sapiens GN=CALM1 PE=1 SV=1
LEGL_HUMAN	Galectin-related protein OS=Homo sapiens GN=LGALSL PE=1 SV=2
CAN1_HUMAN	Calpain-1 catalytic subunit OS=Homo sapiens GN=CAPN1 PE=1 SV=1
HEM2_HUMAN	Delta-aminolevulinic acid dehydratase OS=Homo sapiens GN=ALAD PE=1 SV=1
ARPC4_HUMAN	Actin-related protein 2/3 complex subunit 4 OS=Homo sapiens GN=ARPC4 PE=1 SV=3

S-AgNP

Accession	Protein Name
ALBU_HUMAN	Serum albumin OS=Homo sapiens GN=ALB PE=1 SV=2
CO3_HUMAN	Complement C3 OS=Homo sapiens GN=C3 PE=1 SV=2
TRFE_HUMAN	Serotransferrin OS=Homo sapiens GN=TF PE=1 SV=3
FIBB_HUMAN	Fibrinogen beta chain OS=Homo sapiens GN=FGB PE=1 SV=2
FIBA_HUMAN	Fibrinogen alpha chain OS=Homo sapiens GN=FGA PE=1 SV=2
**APOA1_HUMAN**	**Apolipoprotein A-I OS=Homo sapiens GN=APOA1 PE=1 SV=1**
APOB_HUMAN	Apolipoprotein B-100 OS=Homo sapiens GN=APOB PE=1 SV=2
A1AT_HUMAN	Alpha-1-antitrypsin OS=Homo sapiens GN=SERPINA1 PE=1 SV=3
FIBG_HUMAN	Fibrinogen gamma chain OS=Homo sapiens GN=FGG PE=1 SV=3
HPT_HUMAN	Haptoglobin OS=Homo sapiens GN=HP PE=1 SV=1
CERU_HUMAN	Ceruloplasmin OS=Homo sapiens GN=CP PE=1 SV=1
TLN1_HUMAN	Talin-1 OS=Homo sapiens GN=TLN1 PE=1 SV=3
APOA4_HUMAN	Apolipoprotein A-IV OS=Homo sapiens GN=APOA4 PE=1 SV=3
IGHM_HUMAN	Immunoglobulin heavy constant mu OS=Homo sapiens GN=IGHM PE=1 SV=4
CO4B_HUMAN	Complement C4-B OS=Homo sapiens GN=C4B PE=1 SV=2
TRY1_HUMAN	Trypsin-1 OS=Homo sapiens GN=PRSS1 PE=1 SV=1
ITIH2_HUMAN	Inter-alpha-trypsin inhibitor heavy chain H2 OS=Homo sapiens GN=ITIH2 PE=1 SV=2
ITIH4_HUMAN	Inter-alpha-trypsin inhibitor heavy chain H4 OS=Homo sapiens GN=ITIH4 PE=1 SV=4
IGG1_HUMAN	Immunoglobulin gamma-1 heavy chain OS=Homo sapiens PE=1 SV=1
HEMO_HUMAN	Hemopexin OS=Homo sapiens GN=HPX PE=1 SV=2
FLNA_HUMAN	Filamin-A OS=Homo sapiens GN=FLNA PE=1 SV=4
ANT3_HUMAN	Antithrombin-III OS=Homo sapiens GN=SERPINC1 PE=1 SV=1
FINC_HUMAN	Fibronectin OS=Homo sapiens GN=FN1 PE=1 SV=4

MYH9_HUMAN	Myosin-9 OS=Homo sapiens GN=MYH9 PE=1 SV=4
ACTB_HUMAN	Actin, cytoplasmic 1 OS=Homo sapiens GN=ACTB PE=1 SV=1
VTDB_HUMAN	Vitamin D-binding protein OS=Homo sapiens GN=GC PE=1 SV=1
GELS_HUMAN	Gelsolin OS=Homo sapiens GN=GSN PE=1 SV=1
PLMN_HUMAN	Plasminogen OS=Homo sapiens GN=PLG PE=1 SV=2
CFAB_HUMAN	Complement factor B OS=Homo sapiens GN=CFB PE=1 SV=2
HBA_HUMAN	Hemoglobin subunit alpha OS=Homo sapiens GN=HBA1 PE=1 SV=2
KNG1_HUMAN	Kininogen-1 OS=Homo sapiens GN=KNG1 PE=1 SV=2
APOE_HUMAN	Apolipoprotein E OS=Homo sapiens GN=APOE PE=1 SV=1
HEP2_HUMAN	Heparin cofactor 2 OS=Homo sapiens GN=SERPIND1 PE=1 SV=3
HBD_HUMAN	Hemoglobin subunit delta OS=Homo sapiens GN=HBD PE=1 SV=2
VTNC_HUMAN	Vitronectin OS=Homo sapiens GN=VTN PE=1 SV=1
APOA2_HUMAN	Apolipoprotein A-II OS=Homo sapiens GN=APOA2 PE=1 SV=1
HBB_HUMAN	Hemoglobin subunit beta OS=Homo sapiens GN=HBB PE=1 SV=2
PON1_HUMAN	Serum paraoxonase/arylesterase 1 OS=Homo sapiens GN=PON1 PE=1 SV=3
FETUA_HUMAN	Alpha-2-HS-glycoprotein OS=Homo sapiens GN=AHSG PE=1 SV=1
AACT_HUMAN	Alpha-1-antichymotrypsin OS=Homo sapiens GN=SERPINA3 PE=1 SV=2
IGHA1_HUMAN	Immunoglobulin heavy constant alpha 1 OS=Homo sapiens GN=IGHA1 PE=1 SV=2
IGHG4_HUMAN	Immunoglobulin heavy constant gamma 4 OS=Homo sapiens GN=IGHG4 PE=1 SV=1
CLUS_HUMAN	Clusterin OS=Homo sapiens GN=CLU PE=1 SV=1
IGL1_HUMAN	Immunoglobulin lambda-1 light chain OS=Homo sapiens PE=1 SV=1
IGK_HUMAN	Immunoglobulin kappa light chain OS=Homo sapiens PE=1 SV=1
HRG_HUMAN	Histidine-rich glycoprotein OS=Homo sapiens GN=HRG PE=1 SV=1
C1R_HUMAN	Complement C1r subcomponent OS=Homo sapiens GN=C1R PE=1 SV=2
A1AG2_HUMAN	Alpha-1-acid glycoprotein 2 OS=Homo sapiens GN=ORM2 PE=1 SV=2
APOH_HUMAN	Beta-2-glycoprotein 1 OS=Homo sapiens GN=APOH PE=1 SV=3
APOL1_HUMAN	Apolipoprotein L1 OS=Homo sapiens GN=APOL1 PE=1 SV=5
APOC3_HUMAN	Apolipoprotein C-III OS=Homo sapiens GN=APOC3 PE=1 SV=1
AMBP_HUMAN	Protein AMBP OS=Homo sapiens GN=AMBP PE=1 SV=1
ITA2B_HUMAN	Integrin alpha-IIb OS=Homo sapiens GN=ITGA2B PE=1 SV=3
F13A_HUMAN	Coagulation factor XIII A chain OS=Homo sapiens GN=F13A1 PE=1 SV=4
APOC2_HUMAN	Apolipoprotein C-II OS=Homo sapiens GN=APOC2 PE=1 SV=1
VINC_HUMAN	Vinculin OS=Homo sapiens GN=VCL PE=1 SV=4
PGRP2_HUMAN	N-acetylmuramoyl-L-alanine amidase OS=Homo sapiens GN=PGLYRP2 PE=1 SV=1
IGHG3_HUMAN	Immunoglobulin heavy constant gamma 3 OS=Homo sapiens GN=IGHG3 PE=1 SV=2
1433Z_HUMAN	14-3-3 protein zeta/delta OS=Homo sapiens GN=YWHAZ PE=1 SV=1
TSP1_HUMAN	Thrombospondin-1 OS=Homo sapiens GN=THBS1 PE=1 SV=2

ITB3_HUMAN	Integrin beta-3 OS=Homo sapiens GN=ITGB3 PE=1 SV=2
IGKC_HUMAN	Immunoglobulin kappa constant OS=Homo sapiens GN=IGKC PE=1 SV=2
TPM4_HUMAN	Tropomyosin alpha-4 chain OS=Homo sapiens GN=TPM4 PE=1 SV=3
B3AT_HUMAN	Band 3 anion transport protein OS=Homo sapiens GN=SLC4A1 PE=1 SV=3
ACTN1_HUMAN	Alpha-actinin-1 OS=Homo sapiens GN=ACTN1 PE=1 SV=2
AFAM_HUMAN	Afamin OS=Homo sapiens GN=AFM PE=1 SV=1
TTHY_HUMAN	Transthyretin OS=Homo sapiens GN=TTR PE=1 SV=1
IGHG2_HUMAN	Immunoglobulin heavy constant gamma 2 OS=Homo sapiens GN=IGHG2 PE=1 SV=2
FCN3_HUMAN	Ficolin-3 OS=Homo sapiens GN=FCN3 PE=1 SV=2
APOC1_HUMAN	Apolipoprotein C-I OS=Homo sapiens GN=APOC1 PE=1 SV=1
IGLC2_HUMAN	Immunoglobulin lambda constant 2 OS=Homo sapiens GN=IGLC2 PE=1 SV=1
APOD_HUMAN	Apolipoprotein D OS=Homo sapiens GN=APOD PE=1 SV=1
A1AG1_HUMAN	Alpha-1-acid glycoprotein 1 OS=Homo sapiens GN=ORM1 PE=1 SV=1
C1QB_HUMAN	Complement C1q subcomponent subunit B OS=Homo sapiens GN=C1QB PE=1 SV=3
URP2_HUMAN	Fermitin family homolog 3 OS=Homo sapiens GN=FERMT3 PE=1 SV=1
TBB1_HUMAN	Tubulin beta-1 chain OS=Homo sapiens GN=TUBB1 PE=1 SV=1
PRDX2_HUMAN	Peroxiredoxin-2 OS=Homo sapiens GN=PRDX2 PE=1 SV=5
RAP1B_HUMAN	Ras-related protein Rap-1b OS=Homo sapiens GN=RAP1B PE=1 SV=1
CD5L_HUMAN	CD5 antigen-like OS=Homo sapiens GN=CD5L PE=1 SV=1
TBA4A_HUMAN	Tubulin alpha-4A chain OS=Homo sapiens GN=TUBA4A PE=1 SV=1
**SPTB1_HUMAN**	**Spectrin beta chain, erythrocytic OS=Homo sapiens GN=SPTB PE=1 SV=5**
G3P_HUMAN	Glyceraldehyde-3-phosphate dehydrogenase OS=Homo sapiens GN=GAPDH PE=1 SV=3
TAGL2_HUMAN	Transgelin-2 OS=Homo sapiens GN=TAGLN2 PE=1 SV=3
**SPTA1_HUMAN**	**Spectrin alpha chain, erythrocytic 1 OS=Homo sapiens GN=SPTA1 PE=1 SV=5**
ZPI_HUMAN	Protein Z-dependent protease inhibitor OS=Homo sapiens GN=SERPINA10 PE=1 SV=1
PROF1_HUMAN	Profilin-1 OS=Homo sapiens GN=PFN1 PE=1 SV=2
STOM_HUMAN	Erythrocyte band 7 integral membrane protein OS=Homo sapiens GN=STOM PE=1 SV=3
COF1_HUMAN	Cofilin-1 OS=Homo sapiens GN=CFL1 PE=1 SV=3
ENOA_HUMAN	Alpha-enolase OS=Homo sapiens GN=ENO1 PE=1 SV=2
MYL6_HUMAN	Myosin light polypeptide 6 OS=Homo sapiens GN=MYL6 PE=1 SV=2
KPYM_HUMAN	Pyruvate kinase PKM OS=Homo sapiens GN=PKM PE=1 SV=4
CXCL7_HUMAN	Platelet basic protein OS=Homo sapiens GN=PPBP PE=1 SV=3
SAA4_HUMAN	Serum amyloid A-4 protein OS=Homo sapiens GN=SAA4 PE=1 SV=2
KV320_HUMAN	Immunoglobulin kappa variable 3-20 OS=Homo sapiens GN=IGKV3-20 PE=1 SV=2
S10A8_HUMAN	Protein S100-A8 OS=Homo sapiens GN=S100A8 PE=1 SV=1
ML12A_HUMAN	Myosin regulatory light chain 12A OS=Homo sapiens GN=MYL12A PE=1 SV=2
APOC4_HUMAN	Apolipoprotein C-IV OS=Homo sapiens GN=APOC4 PE=1 SV=1

MOES_HUMAN	Moesin OS=Homo sapiens GN=MSN PE=1 SV=3
CAVN2_HUMAN	Caveolae-associated protein 2 OS=Homo sapiens GN=CAVIN2 PE=1 SV=3
CAP1_HUMAN	Adenylyl cyclase-associated protein 1 OS=Homo sapiens GN=CAP1 PE=1 SV=5
GP1BA_HUMAN	Platelet glycoprotein Ib alpha chain OS=Homo sapiens GN=GP1BA PE=1 SV=2
ILK_HUMAN	Integrin-linked protein kinase OS=Homo sapiens GN=ILK PE=1 SV=2
LDHB_HUMAN	L-lactate dehydrogenase B chain OS=Homo sapiens GN=LDHB PE=1 SV=2
ALDOA_HUMAN	Fructose-bisphosphate aldolase A OS=Homo sapiens GN=ALDOA PE=1 SV=2
PLEK_HUMAN	Pleckstrin OS=Homo sapiens GN=PLEK PE=1 SV=3
CO4A_HUMAN	Complement C4-A OS=Homo sapiens GN=C4A PE=1 SV=2
PLF4_HUMAN	Platelet factor 4 OS=Homo sapiens GN=PF4 PE=1 SV=2
PPIA_HUMAN	Peptidyl-prolyl cis-trans isomerase A OS=Homo sapiens GN=PPIA PE=1 SV=2
HSP7C_HUMAN	Heat shock cognate 71 kDa protein OS=Homo sapiens GN=HSPA8 PE=1 SV=1
GTR14_HUMAN	Solute carrier family 2, facilitated glucose transporter member 14 OS=Homo sapiens GN=SLC2A14 PE=2 SV=1
COR1A_HUMAN	Coronin-1A OS=Homo sapiens GN=CORO1A PE=1 SV=4
TBB5_HUMAN	Tubulin beta chain OS=Homo sapiens GN=TUBB PE=1 SV=2
RSU1_HUMAN	Ras suppressor protein 1 OS=Homo sapiens GN=RSU1 PE=1 SV=3
CATA_HUMAN	Catalase OS=Homo sapiens GN=CAT PE=1 SV=3
TYB4_HUMAN	Thymosin beta-4 OS=Homo sapiens GN=TMSB4X PE=1 SV=2
GP1BB_HUMAN	Platelet glycoprotein Ib beta chain OS=Homo sapiens GN=GP1BB PE=1 SV=1
KV401_HUMAN	Immunoglobulin kappa variable 4-1 OS=Homo sapiens GN=IGKV4-1 PE=1 SV=1
H2B1B_HUMAN	Histone H2B type 1-B OS=Homo sapiens GN=HIST1H2BB PE=1 SV=2
1433E_HUMAN	14-3-3 protein epsilon OS=Homo sapiens GN=YWHAE PE=1 SV=1
1B35_HUMAN	HLA class I histocompatibility antigen, B-35 alpha chain OS=Homo sapiens GN=HLA-B PE=1 SV=1
CAH1_HUMAN	Carbonic anhydrase 1 OS=Homo sapiens GN=CA1 PE=1 SV=2
WDR1_HUMAN	WD repeat-containing protein 1 OS=Homo sapiens GN=WDR1 PE=1 SV=4
KV315_HUMAN	Immunoglobulin kappa variable 3-15 OS=Homo sapiens GN=IGKV3-15 PE=1 SV=2
ANK1_HUMAN	Ankyrin-1 OS=Homo sapiens GN=ANK1 PE=1 SV=3
GSTO1_HUMAN	Glutathione S-transferase omega-1 OS=Homo sapiens GN=GSTO1 PE=1 SV=2
BIN2_HUMAN	Bridging integrator 2 OS=Homo sapiens GN=BIN2 PE=1 SV=3
PDIA3_HUMAN	Protein disulfide-isomerase A3 OS=Homo sapiens GN=PDIA3 PE=1 SV=4
B2MG_HUMAN	Beta-2-microglobulin OS=Homo sapiens GN=B2M PE=1 SV=1
TPIS_HUMAN	Triosephosphate isomerase OS=Homo sapiens GN=TPI1 PE=1 SV=3
GTR1_HUMAN	Solute carrier family 2, facilitated glucose transporter member 1 OS=Homo sapiens GN=SLC2A1 PE=1 SV=2
GPIX_HUMAN	Platelet glycoprotein IX OS=Homo sapiens GN=GP9 PE=1 SV=3
TERA_HUMAN	Transitional endoplasmic reticulum ATPase OS=Homo sapiens GN=VCP PE=1 SV=4
ZYX_HUMAN	Zyxin OS=Homo sapiens GN=ZYX PE=1 SV=1
PGK1_HUMAN	Phosphoglycerate kinase 1 OS=Homo sapiens GN=PGK1 PE=1 SV=3

PRDX6_HUMAN	Peroxiredoxin-6 OS=Homo sapiens GN=PRDX6 PE=1 SV=3
RAB1B_HUMAN	Ras-related protein Rab-1B OS=Homo sapiens GN=RAB1B PE=1 SV=1
PNPH_HUMAN	Purine nucleoside phosphorylase OS=Homo sapiens GN=PNP PE=1 SV=2
VASP_HUMAN	Vasodilator-stimulated phosphoprotein OS=Homo sapiens GN=VASP PE=1 SV=3
1433G_HUMAN	14-3-3 protein gamma OS=Homo sapiens GN=YWHAG PE=1 SV=2
CALR_HUMAN	Calreticulin OS=Homo sapiens GN=CALR PE=1 SV=1
TRFL_HUMAN	Lactotransferrin OS=Homo sapiens GN=LTF PE=1 SV=6
COR1C_HUMAN	Coronin-1C OS=Homo sapiens GN=CORO1C PE=1 SV=1
K2C1_HUMAN	Keratin, type II cytoskeletal 1 OS=Homo sapiens GN=KRT1 PE=1 SV=6
ARF1_HUMAN	ADP-ribosylation factor 1 OS=Homo sapiens GN=ARF1 PE=1 SV=2
CAZA1_HUMAN	F-actin-capping protein subunit alpha-1 OS=Homo sapiens GN=CAPZA1 PE=1 SV=3
PDLI1_HUMAN	PDZ and LIM domain protein 1 OS=Homo sapiens GN=PDLIM1 PE=1 SV=4
EF1A1_HUMAN	Elongation factor 1-alpha 1 OS=Homo sapiens GN=EEF1A1 PE=1 SV=1
RB11A_HUMAN	Ras-related protein Rab-11A OS=Homo sapiens GN=RAB11A PE=1 SV=3
LDHA_HUMAN	L-lactate dehydrogenase A chain OS=Homo sapiens GN=LDHA PE=1 SV=2
CAH2_HUMAN	Carbonic anhydrase 2 OS=Homo sapiens GN=CA2 PE=1 SV=2
GBB1_HUMAN	Guanine nucleotide-binding protein G(I)/G(S)/G(T) subunit beta-1 OS=Homo sapiens GN=GNB1 PE=1 SV=3
TBA1B_HUMAN	Tubulin alpha-1B chain OS=Homo sapiens GN=TUBA1B PE=1 SV=1
CALM1_HUMAN	Calmodulin-1 OS=Homo sapiens GN=CALM1 PE=1 SV=1
LEGL_HUMAN	Galectin-related protein OS=Homo sapiens GN=LGALSL PE=1 SV=2
H4_HUMAN	Histone H4 OS=Homo sapiens GN=HIST1H4A PE=1 SV=2
BLVRB_HUMAN	Flavin reductase (NADPH) OS=Homo sapiens GN=BLVRB PE=1 SV=3
